# TH5487 specifically targets NLRP3 in FCAS patients resistant to MCC950

**DOI:** 10.1038/s42003-026-10008-2

**Published:** 2026-04-16

**Authors:** Angela Lackner, Sofia I. Picucci, Wenjin Jiang, Janset Onyuru, Melissa Campos, Julia E. Cabral, Lemuel Leonidas, Alijah Macapagal, Hannah Lee, Valerie Henriquez, Karen Wang, Huilin Xu, Yanfei Qiu, Lauren V. Albrecht, Hal M. Hoffman, Reginald McNulty

**Affiliations:** 1 Laboratory of Macromolecular Structure, Department of Molecular Biology & Biochemistry, Charlie Dunlop School of Biological Sciences, University of California, Irvine, CA USA; 2 Division of Pediatric Allergy, Immunology, and Rheumatology, Rady Children’s Hospital of San Diego, University of California San Diego School of Medicine, San Diego, CA USA; 3 Department of Developmental and Cell Biology, Charlie Dunlop School of Biological Sciences, University of California, Irvine, CA USA; 4 Department of Pharmaceutical Sciences, University of California, Irvine, CA USA

**Keywords:** Inflammasome, Innate immunity

## Abstract

Aberrant activation of the NLRP3 inflammasome contributes to a wide range of chronic inflammatory disorders. Here, we investigate small-molecule inhibitors originally developed to target the DNA repair enzyme hOGG1 and demonstrate their ability to inhibit NLRP3 activation in human cells. These compounds, including TH5487 (IC50 1.62 µM in human PBMCs), reduce IL-1β secretion while increasing type I interferon responses. Cryo-EM reveals direct association between NLRP3 and mitochondrial DNA, while structural modeling predicts interaction with oxDNA. Notably, inhibitors of the DNA repair glycosylase hOGG1 remain effective in L353P mutant PBMCs from FCAS patients and L351P in mice, at doses where the canonical NLRP3 inhibitor MCC950 is ineffective. Our findings uncover an additional druggable mechanism for inflammasome regulation via interference with oxidized DNA sensing, offering innovative therapeutic opportunities for autoinflammatory disease.

## Abstract (web summary)

Repurposed inhibitors of DNA repair enzyme hOGG1 reduce inflammasome activity, increasing type I interferon responses in human cells, while also inhibiting disease-associated, and gain-of-function NLRP3 in patient PBMCs with autoinflammatory diseases

## Introduction

The NOD-like receptor pyrin domain-containing protein 3 (NLRP3) acts as a pathogen and toxicant sensor, orchestrating inflammasome activation and the maturation and release of IL-1β in response to infection or sterile tissue injury^1,2^. NLRP3 inflammasome activation follows a biphasic process. First, transcriptional priming through bacterial lipoproteins and LPS interaction with TLR2/4, leads to transient NF-κB-mediated upregulation of NLRP3 and pro-IL-1β^3^.

Reactive oxygen species (ROS) production in the mitochondria results in cytidine/uridine monophosphate kinase 2 (CMPK2) initiating mitochondrial replication^4^. The required unraveling of mitochondrial DNA (mtDNA) for replication exposes the DNA to ROS, resulting in oxidation of mtDNA. Repair of oxidized DNA (oxDNA) bases in the mitochondria is performed by human 8-oxoguanine DNA glycosylase 1 (hOGG1)^5^, or elimination of oxidized DNA is conducted through mitophagy^6,7^. Both hOGG1-mediated DNA repair and mitophagy-mediated elimination of oxidized DNA inhibit inflammasome activation in this half of the biphasic process. In the second half, foreign and cytosolic signals such as bacterial toxins, microcrystalline substances, ATP, silica^8^, asbestos, alum, and hydroxyapatite (HA)^9^, overwhelm initial inflammasome inhibition and sustained ROS production, promoting inflammasome subunit assembly and activation. Since most of the agents that initiate inflammasome activation are dissimilar in structure, this suggests that they trigger a common cellular event. One such event is the production of oxidized mitochondrial DNA (ox-mtDNA)^4,10^. Ox-mtDNA is cleaved to 500–650 bp fragments by flap endonuclease 1 (FEN1) to be exported into the cytoplasm via mitochondrial permeability transition pores (mPTP) and voltage-dependent anion channels (VDAC). Once in the cytoplasm, ox-mtDNA can associate with NLRP3, promoting assembly and activation^11^. NLRP3 inflammasome pro-caspase-1 undergoes autocleavage, where mature caspase-1 yields mature IL-1β, IL-18, and gasdermin D. Upon activation, danger-associated molecular patterns (DAMPs), including ox-mtDNA can be released through gasdermin D pores, signaling to other cells via TLRs and promoting additional cytokine release such as TNF.

Even though NLRP3 is broadly involved in innate immunity, there are no FDA-approved inhibitors that target NLRP3 directly. Current treatment for inflammasome-associated pathologies, including cryopyrin-associated periodic syndrome (CAPS), includes Ilaris and Anakinra, which prevent IL-1β from interacting with its receptor. This prevents broad inflammasome activation from any NLR, leaving the cell unable to respond to other pathogens and alarmins. Therefore, there is a clear need for a drug to directly target NLRP3 so the cell can remain responsive to other damage or pathogen-associated molecular patterns (DAMPS/PAMPS). Direct inhibitors of NLR3 have been previously developed, including MCC950, but many have been found to have severe off-target effects and toxicity, leaving the field in need of safe and specific drugs that target NLRP3^12^. Promising new candidates include ZAP-180013, a small molecule that targets the NACHT/LRR interface^13^, but drugs that target the pyrin domain remain unexplored.

NLRP3 has recently been reported to have glycosylase-like activity in that it can cleave oxDNA^14^. The protein fold of the pyrin domain is similar to hOGG1^15,16^, and residues involved in nucleophilic attack of oxDNA in hOGG1 are conserved in NLRP3. Drugs that influence the activity of hOGG1 for the repair of oxDNA also bind to NLRP3, inhibit its interaction with oxDNA, and block inflammasome activation (Fig. S1)^12,14,17,18^. This study aims to decipher the mechanism of inflammasome inhibition by repurposed drugs with hOGG1 activity and examine if they are effective in the context of inflammatory diseases. We illustrate small molecules that bind to NLRP3, prevent inflammasome assembly, and limit the response of NLRP3 to mitochondrial oxidized DNA. Inhibition of NLRP3’s response to oxidized DNA also promotes an alternative cellular response by cGAS-STING and type I interferon (IFN-β). Moreover, we demonstrate that these drugs suppress autoinflammation in the CAPS subtype Familial Cold Autoinflammatory Syndrome (FCAS) PBMCs isolated from patients harboring the NLRP3 L353P mutation, and the mouse equivalent L351P^19^, that are resistant to MCC950^20^.

## Results

### TH5487 and SU0268 inhibit inflammasome activation in primary human PBMCs

Glycosylases are involved in base excision repair, where they remove oxidized guanine from DNA. The recent discovery that NLRP3 has glycosylase activity and shares active site residues with hOGG1 suggests that NLRP3 may interact with similar small molecules as hOGG1^14,15^. Indeed, the hOGG1-targeting small molecule inhibitors TH5487 and SU0268 have been shown to bind to NLRP3 and inhibit inflammasome activation in mouse macrophages^14^. Given the sequence variations between mouse and human NLRP3, which impact disease outcomes differently^21^, we investigated corresponding drug effects in the human cells.

**Fig. 1:**
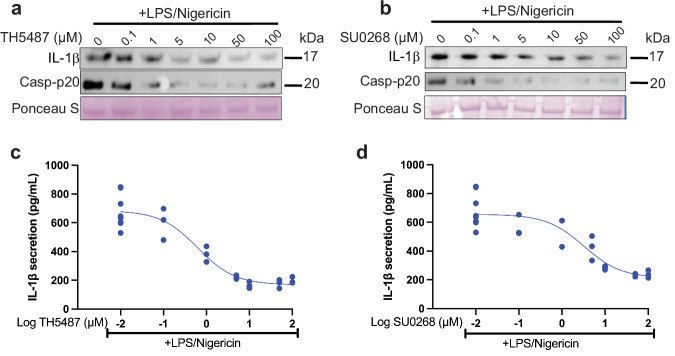
Repurposed hOGG1 inhibitors suppress IL-1β and caspase-1 activation in PBMCs from healthy human donors. **a** Primary human PBMCs from healthy donors were primed with LPS, treated with TH5487 at 0.1–100 µM, then activated with nigericin. IL-1β and Casp-p20 release was visualized by western blot, and Ponceau stain was used as a loading control. **b** Primary human PBMCs were primed with LPS, treated with SU0268 at 0.1–100 µM, then subsequently activated with nigericin. IL-1β and Casp-p20 release was visualized by western blot, and Ponceau stain was used as a loading control. **c** IL-1β release from TH5487-challenged cells was quantified by ELISA. IC50_TH5487_: 1.62 µM, *R*^2^_TH5487_: 0.9203 (*n* = 5 biological replicates). **d** IL-1β release from SU0268-challenged cells was quantified by ELISA. IC50_SU0268_: 3.25 µM, *R*^2^_SU0268_: 0.8209 (*n* = 5 biological replicates). The data were analyzed by nonlinear regression.

Since hOGG1 activation promotes the repair of oxidized mitochondrial DNA^5,22,23^, which subsequently inhibits inflammasome activation^5,11^, selective targeting of hOGG1 by these small molecules would theoretically increase ROS, leading to enhanced inflammasome activation. However, because these molecules also bind to NLRP3, we wanted to confirm whether TH5487 and SU0268 could inhibit inflammasome activation in the complex model of primary human peripheral blood mononuclear cells (PBMCs). These cells from a healthy donor contained a variety of leukocytes commonly found in circulating blood and were selected based on their high percentage of CD14+ monocytes (Table S1). Primary PBMCs were primed for 3 h with 1.6 µg/mL LPS, treated with drugs for 1 h, and activated for 45 min with 20 µM nigericin, and the supernatant of the cells was probed for the inflammasome-associated cytokine IL-1β as well as the cleaved form of Caspase-1 (Fig. 1a, b). We found that TH5487 and SU0268 inhibited IL-1β release. The 1 µM dose of TH5487 inhibited IL-1β secretion by 51.8%, and 5 µM SU0268 inhibited IL-1β and 37.7%, when quantified by ELISA, with an IC50_TH5487_ of 1.68 µM and IC50_SU0268_ of 3.25 µM. (Fig. 1c, d). By probing the lysates of these cells for pro-IL-1β and pro-Caspase-1, we confirmed an inverse relationship, such that greater inhibition by both drugs resulted in increased retention of both pro-IL-1β and pro-Caspase-1 in the cell lysate (Fig. S2). Where we observed almost complete recovery of retained pro-IL-1β in the lysate of TH5487-treated cells compared to LPS-only controls (Fig. S2a, b), the levels of pro-IL-1β retained in the lysate of SU0268-treated cells never recovered to the levels seen in LPS-only controls (Fig. S2c, d). This suggests that SU0268 also plays a role in inhibiting NLRP3 priming, consistent with previous studies^14,18^. Caspase-1 p-20 secretion was also reduced upon treatment with both inhibitors (Fig. S3a, b). Cytotoxicity measurements revealed that these respective doses also reduced overall cell toxicity by 12.6% with 1 µM TH5487 and 11% with 5 µM SU0268 (Fig. S3d, e). We recapitulated this experiment in immortalized human THP-1 monocyte cells, priming them for 16 h with 500 ng/mL LPS and then activating with 20 µM nigericin. As expected, both drugs showed dose-dependent NLRP3 inflammasome inhibition and inhibition of IL-1β, caspase-1 p20, and IL-18 secretion with lower doses of TH5487 and SU0268 than those required in primary PBMCs (Figs. S4 and S5). A 0.1 µM dose of TH5487 and a 1 µM dose of SU0268 inhibited IL-1β secretion by 63.2% and 57.3%, respectively (Fig. S4). We confirmed that these inhibitors work similarly with activation by nigericin or ATP in immortalized mouse macrophages (Fig. S6), and maintain low cytotoxicity from 20 to 28%, consistent with MCC950 treatment in THP-1s (Fig. S7a–d)^24^. NLRP3 inflammasome activation induces both IL‑1β release and pyroptosis, a lytic form of cell death mediated by gasdermin D^25^. In our experiments, hOGG1 inhibitors robustly reduced IL‑1β secretion and caspase‑1 cleavage, yet LDH release remained modest, consistent with partial or sublytic pyroptosis^24,26^. This effect may be further influenced by serum‑free media and the heterogeneous composition of PBMCs, which can limit overt membrane rupture while still allowing inflammasome signaling. Together, these results indicate that the inhibitors suppress inflammasome activation without inducing substantial pyroptotic cell death, highlighting the distinction between cytokine release and terminal lytic events.

To test the NLRP3 specificity of these molecules, we primed NLRP3 knockout THP1s with LPS, treated them with 0–100 µM of TH5487 and SU0268, activated the AIM2 inflammasome by transfecting the cells with non-ox-mtDNA, and evaluated IL-1β secretion by western blot (Fig. S8a). We showed no significant difference in IL-1β secretion, demonstrating NLRP3-specificity in the inhibitory mechanism of the drugs. To further validate our results, we tested other activators and inhibitors. We tested the inhibitory effects of TH5487 and SU0268 in the presence of the activator imiquimod, and the inhibition of the small molecule drug OLT1177, which is currently undergoing clinical trials. We saw similar inhibitory effects in THP1 cells activated with imiquimod (Fig. S8d–f) and robust inhibition in THP1 cells challenged with OLT1177 (Fig. S8g, h).

### SU0268 and TH5487 prevent assembly of NLRP3 inflammasome subunits

To further investigate the mechanism of inhibition, we examined the effect of these drugs on inflammasome assembly. We hypothesized that these small molecules would interfere with the formation of the NLRP3 inflammasome. We stimulated human monocytes (THP-1s) in the presence of LPS/nigericin, along with increasing concentrations of SU0268 or TH5487. Co-immunoprecipitation against NLRP3 was then performed to assess assembly of key inflammasome subunits: pro-caspase-1, NEK7, and ASC. Co-immunoprecipitated proteins were quantified by normalizing the band intensities of pro-caspase-1, NEK7, and ASC to the corresponding NLRP3 band.

**Fig. 2:**
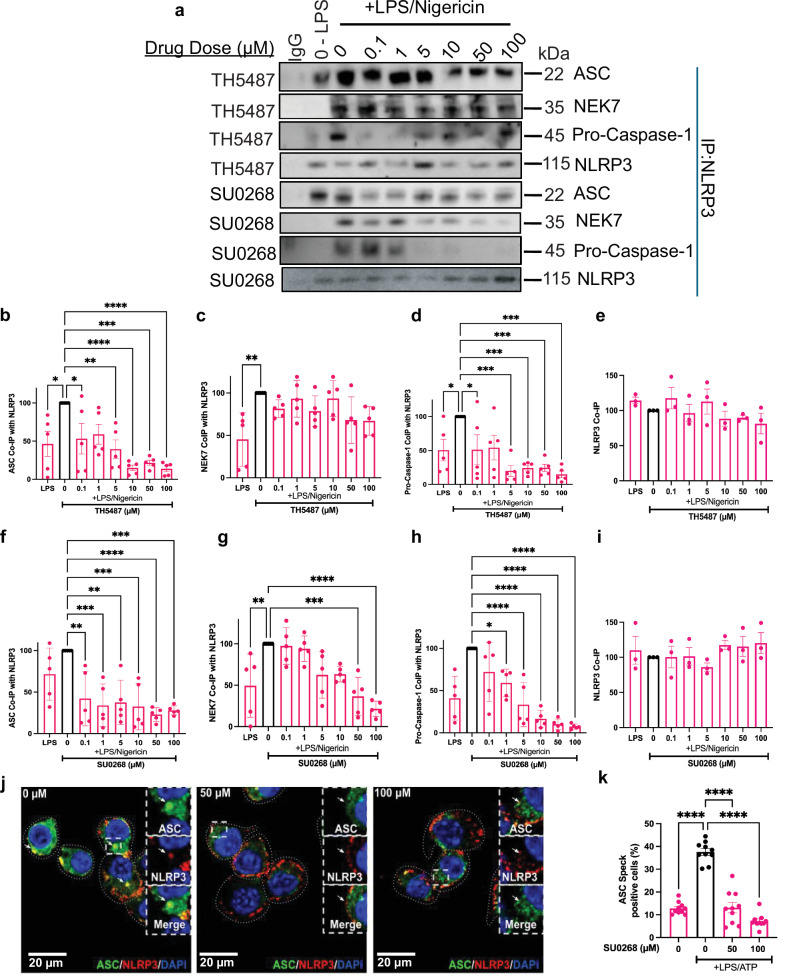
TH5487 and SU0268 disrupt inflammasome assembly by blocking NLRP3-dependent recruitment of ASC, NEK7, and pro-caspase-1. **a** THP1 cells were primed with LPS, treated with either TH5487 or SU0268 at 0.1–100 µM, and activated with nigericin. NLRP3 was isolated using protein G magnetic beads, and proteins that co-immunoprecipitated with NLRP3 were visualized by Western blots. The following graphs represent the quantified amount of ASC (**b**) NEK7, (**c**) and Pro-caspase-1 (**d**) that co-immunoprecipitated with NLRP3 (**e**) in primed, activated, and TH5487-inhibited cells. The following graphs represent the quantified amount of ASC (**f**), NEK7 (**g**), and Pro-caspase-1 (**h**) that co-immunoprecipitated with NLRP3 (**i**) in primed, activated, and SU0268-inhibited cells (**a**–**i**, *n* = 3-5 biological replicates). **j** ASC speck formation was visualized by immunofluorescence. Immortalized BMDMs were primed with LPS and activated with ATP. ASC was stained green, NLRP3 was stained red, and the nucleus was stained blue. Left: No drug treatment (arrow points to an example of an ASC speck), Middle: 50 µM SU0268 treatment, Right: 100 µM SU0268 treatment. **k** Quantification of the percentage of ASC speck-containing cells from (**h**), where >1000 cells per condition were analyzed. For all graphs, error bars signify the mean ± SEM. The data was analyzed by one-way ANOVA. p**** < 0.0001, p*** < 0.001, p** < 0.01, p* < 0.05.

We found 0.1 µM TH5487 was sufficient to reduce the amount of ASC associated with NLRP3 by 77.3% (Fig. 2a, b). The ser/thr kinase NEK7 showed an increase upon addition of nigericin alone, but no significant difference in NEK7 association with NLRP3 was observed with 0.1–100 µM TH5487 (Fig. 2a, c). Pro-caspase-1 decreased by 78.2% with 5 µM TH5487 (Fig. 2a, d). The same amount of lysate was loaded for each treatment, showing no change in NLRP3 levels (Fig. 2e). Concentrations as low as 0.1 µM SU0268 caused 81.3% reduction in ASC association with NLRP3 (Fig. 2a, f). Unlike TH5487, 10 µM SU0268 decreased NEK7 association by 35.7% (Fig. 2a, g). SU0268 outperformed TH5487 in preventing association with pro-caspase-1. A 46% reduction in pro-caspase-1 occurred with 0.1 µM SU0268 (Fig. 2a, h). The observed decrease in NEK7 binding, which can interact with NLRP3 independently of ASC, highlights the specificity of the drugs in affecting proteins that directly bind NLRP3 within the inflammasome complex and related partners. We further validated this data using immunofluorescence staining to show that both 50 and 100 µM of SU0268 efficiently inhibited the association between NLRP3 and ASC compared to control cells, and confirmed that these drugs inhibited ASC speck formation, a hallmark of NLRP3 inflammasome activation (Figs. 2j and S9)^27,28^. Treatment with 50 µM SU0268 reduced ASC speck formation by 75.8% compared to controls, and 100 µM SU0268 further reduced ASC speck formation by 90.6% (Fig. 2k). We did not detect a decrease in NLRP3 or ASC protein expression in response to the SU0268 treatment (Fig. S10a). We confirmed that the expression of NLRP3, ASC, or hOGG1 proteins was not affected by treatment of SU0268 from 0.1–100 µM by western blot and RT-qPCR (Fig. S10b–f). These results indicate that the drugs impact NLRP3 interactions with both oxidized DNA^14^ and inflammasome subunits, confirming the link between NLRP3’s oxidized DNA interactions and its ability to form an active inflammasome complex.

### Dual inhibition of NLRP3 and hOGG1 by TH5487 and SU0268

Since TH5487 and SU0268 also directly inhibit hOGG1^17,18^, and since hOGG1 can increase NF-kB activity^29,30^, we hypothesized these drugs might limit the production of priming cytokines like TNF-α^33^. Indeed, we found that TNF-α expression, slightly elevated with LPS/ATP treatment, was reduced in the presence of TH5487 or SU0268 (Fig. S11a, b). A concentration of 5 µM TH5487 reduced TNF-α expression by 21% (Fig. S11a). Similarly, 5 µM SU0268 reduced TNF-α expression by 35% compared to LPS/ATP treatments (Fig. S11b). To explore if TNF-α secretion was reduced in these conditions, cells were primed with LPS, challenged with TH5487 or SU0268, and subsequently activated with ATP. Then the supernatant was probed using a western blot for TNF-α secretion (Fig. S11c). A concentration of 0.1 µM TH5487 reduced TNF-α release by 35.2%. (Fig. S11d). Similarly, 0.1 µM SU0268 reduced TNF-α release by 25.9% compared to LPS/ATP treatments (Fig. S11e). A higher dose of 50 µM TH5487 reduced TNF-α secretion further by 55.6%. However, a more sensitive response was achieved with 5 µM SU0268, which caused TNF-α reduction by 45.5%. Both drugs inhibit TNF-α production and signaling, but lower concentrations of SU0268 bring about higher inhibitory effects. Our data support previous studies showing TH5487 and SU0268 inhibit LPS-induced TNF-α, due to their ability to inhibit both hOGG1 (affecting priming through NF-κB) and NLRP3 (affecting activation)^17,18^. To define the contribution of priming inhibition to the NLRP3 inhibition we observe, we evaluated IL-1β secretion when inhibitors were added at the same time as the activation phase, compared to when they were added 1 h before. We observed minimal reduction in efficacy when bypassing the priming phase, suggesting that effects on priming pathways contribute minimally to the overall inhibitory activity at the concentrations tested (Fig. S11f, g)

### TH5487 and SU0268 inhibit NLRP3 activation and mitochondrial association in response to mitochondrial dysfunction

**Fig. 3:**
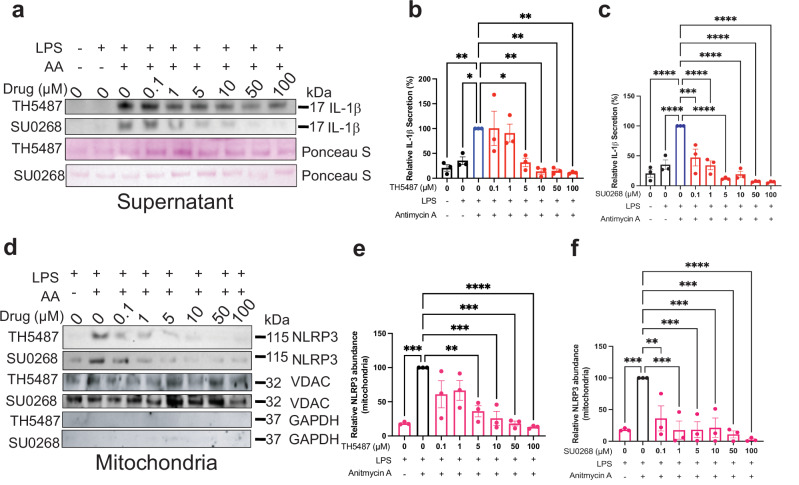
Repurposed inhibitors block IL-1β release and NLRP3 mitochondrial localization in response to mitochondrial ROS. **a** THP1 cells were primed with LPS, treated with either TH5487 or SU0268 at 0.1–100 µM, and activated with Antimycin A. Relative IL-1β release was visualized by western blot, and Ponceau stain was used as a loading control. **b** Quantification of (**a**) for TH5487 (*n* = 3 biological replicates). **c** Quantification of (**a**) for SU0268 (*n* = 3 biological replicates). **d** THP1 cells were primed with LPS, treated with either TH5487 or SU0268 at 0.1–100 µM, and activated with Antimycin A. The mitochondrial fraction was isolated, and the relative amount of NLRP3 associated with the mitochondria was visualized by western blot. **e** Quantification of (**d**) for TH5487 (*n* = 3 biological replicates). **f** Quantification of (**d**) for SU0268 (*n* = 3 biological replicates). For all graphs, error bars signify the mean ± SEM. The data were analyzed by one-way ANOVA. p**** < 0.0001, p*** < 0.001, p** < 0.01, p* < 0.05.

Antimycin A (AA) inhibits mitochondrial complex III, leading to electron transport chain disruption and increased production of mitochondrial ROS^31^. Mitochondrial membrane potential perturbations are known to promote the release of ox-mtDNA and induce NLRP3 inflammasome activation. Prior studies, including those by the Subramanian group, have shown that NLRP3 associates with the mitochondria via the mitochondrial antiviral signaling protein (MAVS)^32^. To test if NLRP3 activation driven by mitochondrial stress could be inhibited by repurposed glycosylase small molecules, we exposed immortalized THP-1 cells to 500 ng/mL LPS for 16 h, treated with TH5487 or SU0268, then induced ROS production with 10 µM AA for 2 h. AA robustly induced IL-1β secretion, which was suppressed by TH5487 and SU0268 (Fig. 3a). Specifically, 5 µM TH5487 and 0.1 µM SU0268 reduced IL-1β secretion by 68.2% and 52.8%, respectively (Fig. 3b, c). Furthermore, AA promoted NLRP3 localization to mitochondria, and this mitochondrial translocation was markedly reduced by both inhibitors (Fig. 3d). Concentrations as low as 5 µM TH5487 reduced NLRP3 mitochondrial localization by 63.9%, and 0.1 µM SU0268 reduced NLRP3 mitochondrial localization by 70% (Fig. 3e, f). Importantly, total cytosolic NLRP3 levels were not significantly altered by these treatments (Fig. S12), suggesting that a small, regulated pool of NLRP3 is targeted to mitochondria upon activation. These findings support previous data showing ox-mtDNA is sufficient to induce inflammasome activation^4^ and NLRP3 recruitment to the mitochondria^32,33^. These inhibitors disrupt both inflammasome activation and NLRP3 mitochondrial localization in response to mitochondrial ROS.

### Inhibiting NLRP3 interaction with ox-mtDNA promotes compensation by cGAS-STING

Stimulation of monocytes with LPS/ATP or LPS/nigericin leads to alterations in the membrane potential of the outer membrane and within intracellular organelles, including the ER, Golgi, and mitochondria^34,35^. These hyperpolarization changes at the outer membrane induce ionic fluxes in intracellular organelles, resulting in abnormal fluctuations in sodium, potassium, calcium, and other signaling molecules^36–38^. Although TH5487 and SU0268 have been shown to decrease the release of ox-mtDNA^14^, complete inhibition of NLRP3 leads to a cytosolic increase of DNA that is not mitochondrial in origin^14^.

**Fig. 4:**
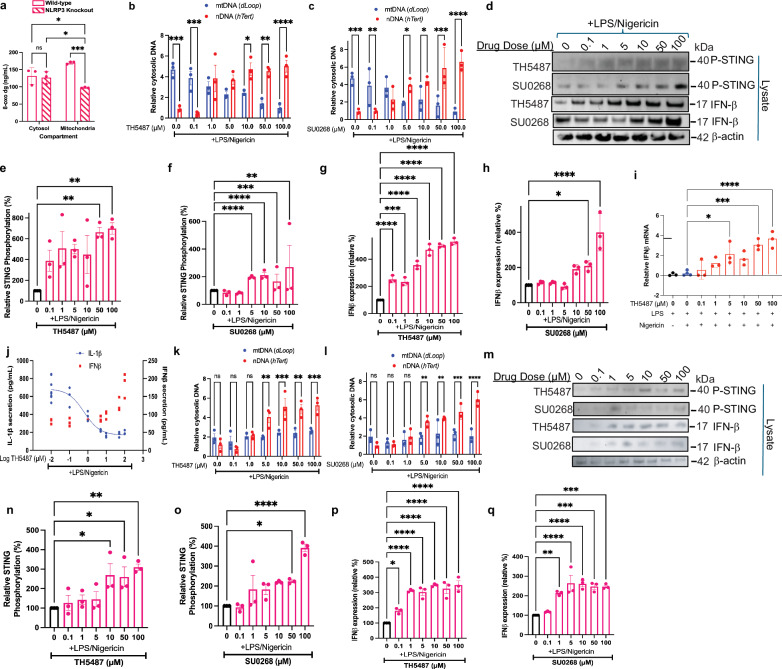
NLRP3 inhibition promotes cGAS-STING activation and IFN-β production in primary human PBMCs. **a** NLRP3 wild type and knockout cells were treated with LPS and ATP, the mitochondrial and cytosolic fractions were isolated, and the amount of oxidized DNA in each compartment was quantified by and 8-oxo-dG ELISA. Primary human PBMCs were activated with LPS and nigericin and were treated with TH5487 (**b**) or SU0268 (**c**) at 0.1–100 µM. The cytosolic, mitochondrial, and nuclear fractions of DNA were isolated, and qPCR was run against *hTert* as a nuclear probe and *D-loop* as a mitochondrial probe. The ratio of *hTert/D-loop* in the cytosol to *hTert/D-loop* in the nucleus/mitochondria (respectively) was analyzed by two-way ANOVA. **d** THP1 cells activated with LPS and nigericin were treated with either TH5487 or SU0268 at 0.1–100 µM. The relative amount of internal phosphorylated STING (**e**, **f**) or IFN-β expression (**g**, **h**) was quantified for both drugs by one-way ANOVA. **i** Primary human PBMCs were activated with LPS and nigericin and treated with TH5487 at 0.1–100 µM, and IFN-β expression was quantified with qPCR. **j** Primary human PBMCs were activated with LPS and nigericin and treated with TH5487 at 0.1–100 µM. IFN-β (red) and IL-1β b (blue) release was quantified by ELISA. Data were analyzed using nonlinear regression: IC50_TH5487_: 1.62 µM, *R*^2^_TH5487_: 0.9203. NLRP3 knockout THP1s were activated with LPS and nigericin and were treated with TH5487 (**k**) or SU0268 (**l**) at 0.1–100 µM. The cytosolic, mitochondrial, and nuclear fractions of DNA were isolated, and qPCR was run against *hTert* as a nuclear probe and *D-loop* as a mitochondrial probe. The ratio of *hTert/D-loop* in the cytosol to *hTert/D-loop* in the nucleus/mitochondria (respectively) was analyzed by two-way ANOVA. **m** NLRP3 knockout THP1 cells activated with LPS and nigericin were treated with either TH5487 or SU0268 at 0.1–100 µM. The relative amount of internal phosphorylated sting (**n**, **o**) or IFN-β expression (**p**, **q**) was quantified for both drugs by one-way ANOVA. For all bar graphs, error bars signify the mean ± SEM with *n* = 3 biological replicates, where p**** < 0.0001, p*** < 0.001, p** < 0.01, p* < 0.05.

We hypothesize that these significant ionic fluxes within the cell could eventually lead to disturbances and the secretion of nuclear DNA, which may become oxidized and released into the cytosol. Thus, inhibition of NLRP3 is expected to increase cytosolic DNA levels, specifically from nuclear, not mitochondrial, sources. Presence of oxidized and non-oxidized DNA in the cytosol could subsequently activate the cGAS-STING pathway^39,40^. We found that NLRP3^−/−^ have decreased oxidized DNA in the mitochondrial compartment compared to wildtype. However, there was no significant difference in oxidized DNA released to the cytosol for NLRP3^−/−^ compared to wildtype (Fig. 4a). This suggests that, in response to a loss of NLRP3, the amount of cytosolic oxidized DNA may be compensated by DNA that is of nuclear origin. We validated this by measuring nuclear DNA content in the cytosol, nucleus, and mitochondria of primary human PBMCs that were primed for 3 h with 1.6 µg/mL LPS, challenged with either TH5487 or SU0268, and activated for 45 min with 20 µM nigericin. Both inhibitors reduced the amount of cytosolic mitochondrial DNA while simultaneously increasing the amount of cytosolic nuclear DNA in a concentration-dependent manner. TH5487 caused an 82.1% increase in nuclear DNA (nDNA), and a 78.7% decrease in mtDNA at 100 µM compared to controls, such that at 0 µM TH5487, the ratio of mtDNA to nDNA was 5:1, and 1:5 at 100 µM (Fig. 4b). Similarly, SU0268 caused an 86.4% increase in nDNA, and an 80.9% decrease in mtDNA at 100 µM compared to controls, such that at 0 µM SU0268, the ratio of mtDNA to nDNA was 5:1, and 1:7 at 100 µM (Fig. 4c). We then checked for cGAS-STING activity, which may respond to the increase in cytosolic nuclear DNA when NLRP3 is inhibited in the presence of LPS/nigericin. Indeed, we observed a concentration-dependent increase in phosphorylated STING (p-STING) with escalating levels of TH5487 or SU0268 in human THP1 cells, confirming that NLRP3 inhibition triggers cGAS-STING activation (Fig. 4d). The amount of p-STING increased by 43% with 1 µM TH5487 (Fig. 4e), while 5 µM of SU0268 increased the amount of p-STING by 93% (Fig. 4f). Activation of cGAS-STING is known to lead to a type-1 interferon response^40–42^. We found 0.1 µM TH5487 caused a 107.5% increase in cytosolic IFN-β when stimulated with LPS/nigericin (Fig. 4g). Less sensitivity was seen with 50 µM SU0268, which caused a 101.9% increase in IFN-β (Fig. 4h). To determine whether these findings extend to a more physiological model, we repeated experiments using PBMCs. We first evaluated IFN-β expression by RT-qPCR across a range of drug concentrations (0.1–100 µM). TH5487 and SU0268 increased IFN-β transcript levels by 10.7% (5 µM) and 7% (10 µM), respectively (Figs. 4i and S13a, c). To assess whether this transcriptional upregulation translated to IFN-β secretion, we performed ELISA assays on the supernatant. Treatment with 50 µM TH5487 and SU0268 increased IFN-β secretion by 1.6-fold and 3.7-fold, respectively (Figs. 4j and S13b, d), confirming that inhibition of NLRP3 with these repurposed OGG1 inhibitors promotes type I interferon responses through the cGAS-STING axis. To examine if the cGAS activation we observe is NLRP3 independent, we ran the same assays in NLRP3 knockout cells. We first evaluated mtDNA and nDNA levels in activated NLRP3 KO cells in the presence of 0.1–100 µM TH5487 or SU0268. We observed a similar increase in cytosolic nDNA in response to the drugs as with the wild-type cells, but overall lower and sustained levels of cytosolic mtDNA (Fig. 4k, l). This supports our previous data suggesting NLRP3 plays some role in mtDNA release into the cytosol^14^. We also evaluated cytosolic P-STING and IFN-β levels the same way we did in the wild-type cells. We showed a similar increase in both STING phosphorylation and IFN-β expression as we did in the wild-type cells, suggesting that the cGAS activation we observe is NLRP3 independent (Fig. 4m–q).

### Biophysical analysis reveals NLRP3 binds mitochondrial DNA

**Fig. 5:**
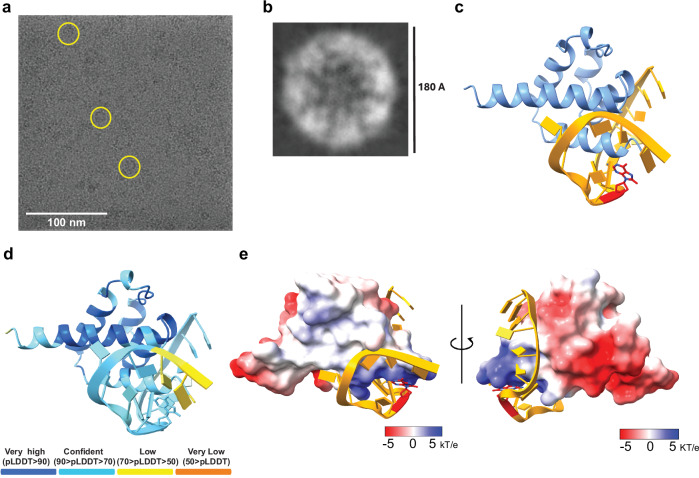
NLRP3 binds oxidized mitochondrial DNA revealed by cryo-EM. **a** Example cryo-EM micrograph after streptavidin lattice removal. Yellow circles show NLRP3 particles bound to the streptavidin/biot-DNA layer. **b** Sample class average measures ~18 nm (180 Å) in diameter, similar to the size of the published inactive decamer of NLRP3 (PDB 7PZC). **c** An AlphaFold-3 model of the NLRP3 pyrin domain (residues 1–85, blue) interacting with single-stranded (orange) oxidized DNA (red, from PDBID: 1EBM). **d** The color-coded confidence prediction from (**c**). PTM = 0.79 iPTM = 0.73. **e** The electrostatic potential of the NLRP3 pyrin domain AlphaFold model shows a more negative (blue) charge in the region closest to the oxidized base (red) in the single-stranded DNA (orange). The model has been rotated horizontally 90° to show two different views.

Given our in vitro data supporting the functional relevance of inhibiting NLRP3:DNA interactions, we sought to further define this relationship biophysically. We adapted a method from the Nogales group using streptavidin-coated affinity cryo-electron microscopy (cryo-EM) grids^43^. Full-length NLRP3 was first incubated with biotinylated, non-oxidized mitochondrial DNA (biot-DNA) to form a stable NLRP3–biotin-DNA complex, which was then applied to the streptavidin-coated grid surface (Fig. S14a). Non-oxidized DNA was used because it binds NLRP3 but is not cleaved^14^. NLRP3 was purified as a decamer in complex with TH5487 and confirmed as a single high-order species by SDS-PAGE, western blot, and native gel electrophoresis (Fig. S14b, c). After applying the NLRP3–biot-DNA complex to the affinity grids, particles were visualized using a Titan Krios microscope equipped with a Falcon 4i detector. While particles of various sizes were present, the underlying streptavidin lattice was visible in the background. Since the Fourier transform of a lattice is another lattice, the reciprocal lattice spots were visible in the FFT (Fig. S14d, e). Computational subtraction of the streptavidin lattice markedly improved particle visualization and eliminated FFT contamination (Fig. S14f)^44^. This revealed two main populations of particles: a smaller class around 100 Å and a larger class between 180–200 Å (Fig. 5a). Class averages revealed a major population of particles at 180 Å, consistent with the published cryo-EM structure of NLRP3 bound to the small-molecule inhibitor MCC950^45^ (Fig. 5b).

To examine if the NLRP3 pyrin domain alone is predicted to interact with oxidized DNA, we used AlphaFold2 to show that single-stranded (ss) oxDNA could bind the pyrin domain (Fig. 5c). This interaction was predicted with high confidence (PTM = 0.79, iPTM = 0.73) and localizes near a positively charged patch of the pyrin domain (Fig. 5d, e). Interestingly, non-oxidized ssDNA or double-stranded oxDNA is not predicted to interact with the pyrin domain, instead yielding two models far apart from one another (Fig. S15a–c). In the context of full monomeric NLRP3, the ss oxDNA is predicted to localize near the NACHT/LRR domain (Fig. S15d), consistent with our previous data showing that NLRP3 can bind oxDNA both in the pyrin domain and the NACHT-LRR^46^.

### hOGG1 activator TH10785 inhibits NLRP3 inflammasome activation in primary human PBMCs

**Fig. 6:**
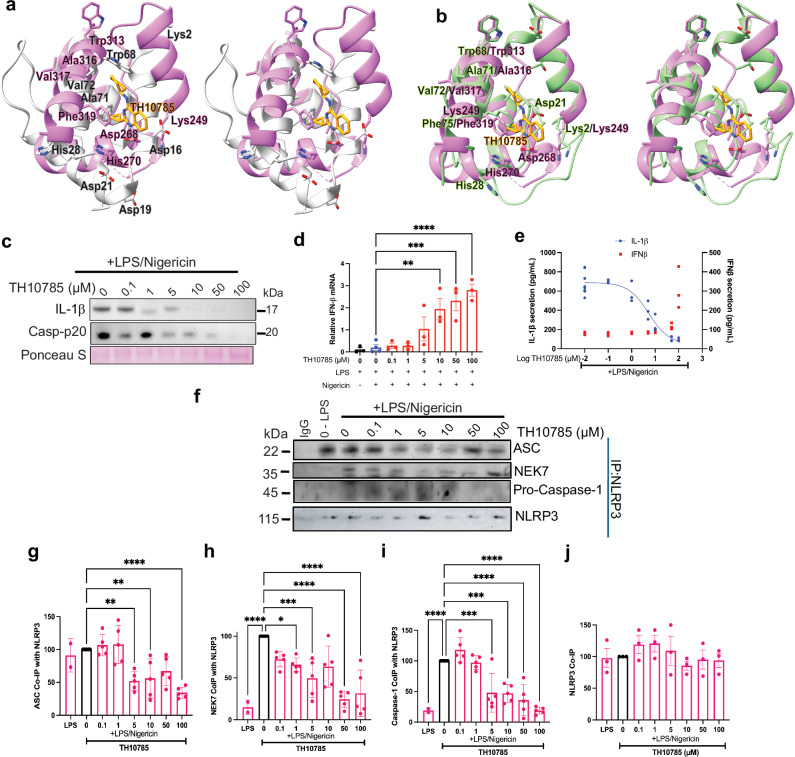
TH10785, a known hOGG1 activator, unexpectedly inhibits NLRP3 and promotes interferon signaling. **a** The NLRP3 pyrin domain (gray, PDBID: 7PZC) docked into the structure of hOGG1 bound to TH10785 (pink, PDBID: 7AYY). Left: labeled homologous amino acids, right: unlabeled structures. **b** SWISS-MODEL projection of NLRP3 pyrin based on hOGG1 bound to ox-DNA (green) docked into the structure of hOGG1 bound to TH10785 (pink, PDBID: 7AYY). Left: labeled homologous amino acids, right: unlabeled structures. **c** Primary human PBMCs were primed with LPS, and treated with TH10785 at 0.1–100 µM, and activated with nigericin. IL-1β and Casp-p20 release was visualized by western and Ponceau stain was used as a loading control **d** IFN-β expression was quantified with qPCR (*n* = 3 biological replicates). **e** IFN-β (red) and IL-1β (blue) release were quantified by ELISA. Error bars signify the mean ± SD. IC50_TH10785_: 5.329 µM, *R*^2^_TH10785_: 0.9031. **f** THP1 cells activated with LPS and ATP were treated with either TH10785 at 0.1–100 µM. NLRP3 was isolated using protein G magnetic beads, and western blots on the fraction that co-immunoprecipitated with NLRP3 were run. The following graphs represent the quantified amount of ASC (**g**) NEK7 (**h**) and Pro-caspase-1 (**i**) that co-immunoprecipitated with NLRP3 (**j**) (*n* = 5 biological replicates). For bar graphs, error bars signify the mean ± SEM, and the data were analyzed by one-way ANOVA, where p**** < 0.0001, p*** < 0.001, p** < 0.01, p* < 0.05.

The drugs TH5487 and SU0268 inhibit hOGG1, and we demonstrate herein that they also inhibit NLRP3. From a therapeutic perspective, inhibiting hOGG1 could be beneficial by allowing an increase in cytosolic oxidized DNA, which may enhance the type-1 interferon immune response. Conversely, if reducing an overactive immune response is desirable, promoting DNA repair by activating hOGG1 while simultaneously inhibiting NLRP3 inflammasome activation could be advantageous. Given that TH5487 and SU0268 inhibit hOGG1, we sought a drug capable of activating hOGG1. TH10785 (Fig. S2e) acts as an hOGG1 activator, initiating DNA repair to mitigate DNA damage and reduce ROS production^47^. So, we investigated whether TH10785 could inhibit NLRP3 activation and inflammasome assembly. Superposition of NLRP3 pyrin and hOGG1 bound to TH10785 showed a similar binding pocket for TH10785 (Fig. 6a). The model was further improved using SWISS MODEL (Fig. 6b). Homologous residues critical for catalytic activity in hOGG1 show improved alignment in the model, with key residues (NLRP3 Lys3, Asp21, and Phe75) moving within 0.6–1.0 Å of their hOGG1 counterparts (Table S2). Next, we tested whether TH10785 could inhibit NLRP3 inflammasome activation in primary human PBMCs primed with LPS, challenged with 0.1–100 µM of TH10785 and activated with nigericin. We probed the supernatant for IL-1β and Caspase-p20 release (Fig. 6c). Concentrations as low as 5 µM TH10785 inhibited NLRP3-dependent IL-1β secretion by 41.4% when quantified by ELISA (Fig. 6d). Pro-IL-1β and pro-Caspase-1 levels retained intracellularly were increased (Fig. S2e, f), while Caspase-p20 secretion was also reduced (Fig. S3c). TH10785 also restores intercellular pro-Caspase-1 and pro-IL-1β levels in THP1 cells (Fig. S5e, f). We conducted the same experiments to evaluate cGAS-STING pathway activation in cells treated with TH10785. We saw a 9.5-fold increase in IFN-β expression by RT-qPCR with 10 µM TH10785, and a 3.8-fold increase in IFN-β secretion via ELISA with 100 µM TH10785 (Fig. 6d, e). This supports cGAS-STING inflammatory compensation in response to NLRP3 inhibition, which may be partially due to increased cytotoxicity of the drug at high doses (Fig. S3f).

### TH10785 inhibits NLRP3 inflammasome activation and assembly in monocytes

To test if inflammasome activity could be inhibited by TH10785 in human monocytes, THP-1 cells were primed with LPS, treated with 0.001–100 µM TH10785, and activated with nigericin. We evaluated overall inflammasome activation by measuring IL-1β and Caspase-p20 secretion (Fig. S2f and S16a, b). Concentrations as low as 0.1 µM of TH10785 inhibited NLRP3-dependent IL-1β secretion by approximately 50% (Fig. S16b). Similar results were seen in mouse iBMDM cells activated with LPS and ATP and treated with TH10785 (Fig. S16c–f). To examine the mechanism of TH10785-mediated NLRP3 inhibition, we performed a co-IP of NLRP3 with ASC, pro-caspase-1, and NEK7 (Figs. 6f and S17). We found that 5 µM TH10785 decreased the association of ASC, pro-caspase-1, and NEK7 by 47.7%, 38.2%, and 67.4%, respectively (Fig. 6g–j). These results are significant because they illustrate that we can decrease hOGG1 activity with TH5487 and SU0268, or increase hOGG1 activity with TH10785, while simultaneously inhibiting inflammasome activation.

### TH5487 inhibits IL-1β secretion from disease-associated FCAS mouse and human primary cells

**Fig. 7:**
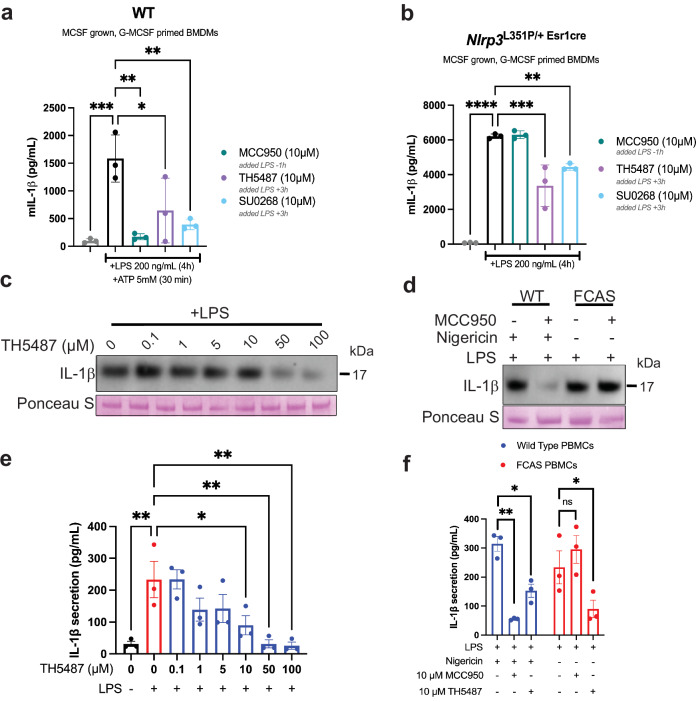
Repurposed inhibitors block inflammasome activation in FCAS BMDMs and PBMCs resistant to MCC950. **a** BMDMs isolated from wild-type mice were primed with LPS, challenged with MCC950, TH5487, or SU0268, then activated with ATP. IL-1β secretion was quantified by ELISA. **b** BMDMs isolated from L351P CAPS mice were primed with LPS and challenged with MCC950, TH5487, or SU0268. IL-1β secretion was quantified by ELISA. **c** Primary human PBMCs from patients harboring the L353P FCAS CAPS mutation were activated with LPS and treated with TH5487 at 0.1–100 µM. IL-1β release was visualized by Western blot, and Ponceau stain was used as a loading control. **d** Primary human PBMCs from healthy donors and patients harboring the L353P CAPS mutation were activated with LPS (CAPS) or LPS and nigericin (healthy) and treated with TH5487 or MCC950 at 10 µM. IL-1β release was visualized by Western blot, and Ponceau stain was used as a loading control. **e** IL-1β release from the L353P cells challenged with the dose titration of TH5487 was quantified by ELISA. **f** IL-1β release from the healthy or L353P cells challenged with 10 µM TH5487 or MCC950 was quantified by ELISA. For both graphs, error bars signify the mean ± SEM, and the data were analyzed by one-way ANOVA with *n* = 3 biological replicates, where p**** < 0.0001, p*** < 0.001, p** < 0.01 and p* < 0.05.

Given the need for effective NLRP3 inhibitors in autoinflammatory diseases, we tested the ability of TH5487 to inhibit NLRP3-dependent IL-1β secretion in primary mouse BMDMs from mice harboring the FCAS L351P mutation, and primary PBMCs isolated from patients harboring the human equivalent L353P FCAS mutation. This mutation causes the FCAS subtype of CAPS, characterized by cold-induced inflammasome hyperactivation and IL-1β overproduction^1^. We sought to compare our results to the well-characterized NLRP3 inhibitor MCC950, which is ineffective at blocking NLRP3 activity in CAPS patients with the L353P mutation^20^. Wild-type mouse BMDMs were primed and activated with LPS and ATP, and challenged with either 10 µM MCC950, 10 µM TH5487, or 10 µM SU0268. Treatment with all drugs significantly inhibited IL-1β secretion from wild-type BMDMs (Fig. 7a). However, in BMDMs isolated from mice harboring FCAS L351P, primed with LPS, and treated with all 3 drugs, only TH5487 and SU0268 significantly reduced IL-1β secretion (Fig. 7b), highlighting the unique ability for these repurposed drugs to function in difficult-to-treat monogenic NLRP3-mediated diseases. To further evaluate relevance in a disease-relevant cellular context of these inhibitors, primary human PBMCs from FCAS L353P patients were primed for 3 h with 1.6 µg/mL LPS and treated with TH5487 at 0.1–100 µM for 1 h, and IL-1β release was visualized by western blot (Fig. 7c). Since CAPS mutants can harbor an inflammatory response without a secondary activating signal, no ATP or nigericin was added to this study. To compare TH5487 efficacy to MCC950, wild-type and FCAS PBMCs were primed and activated as previously described, treated with 10 µM TH5487 or MCC950, and IL-1β release was visualized by western blot (Fig. 7d). IL-1β secretion from L353P cells was decreased by 61.4% upon treatment with 10 µM TH5487 (Fig. 7e). In the FCAS PBMCs, 10 µM MCC950 proved ineffective, but in wild-type PBMCs, 10 µM MCC950 and 10 µM TH5487 both inhibited NLRP3-dependent IL-1β release by 82.5% and 48.6%, respectively (Fig. 7f). These findings highlight the therapeutic potential of this innovative inhibitor to overcome MCC950 resistance in FCAS.

## Discussion

In summary, this study reveals that inhibitory or activating drugs that bind human glycosylase hOGG1 can be repurposed to target NLRP3 inflammasome activation in physiologically relevant peripheral blood mononuclear cells (PBMCs) isolated from human blood. Moreover, FCAS patients with L353P that do not respond to MCC950 respond to TH5487 (Fig. 7). In the severe mouse model of FCAS, L351P BMDMs were unresponsive to 10 mM MCC950, but were responsive to our repurposed inhibitors (Fig. 7). MCC950 inhibits NLRP3 inflammasome activation with an IC₅₀ of approximately 7.5 nM in primary macrophages^26^. In comparison, we observe IC₅₀ values of 1.6, 3.25, and 5.3 µM for TH5487, SU0268, and TH10785, respectively, in PBMCs, consistent with their distinct and dual mechanisms of OGG1 and NLRP3 inhibition. These repurposed inhibitors stabilize NLRP3 and support an interaction with the pyrin domain^14^, resulting in inhibition of inflammasome activation in the presence of Antimycin A (Fig. 3). If the inhibitory response was not NLRP3-dependent, one would expect an increase in inflammasome activation when ROS and oxDNA are generated when AA inhibits the mitochondrial electron transport chain, especially with hOGG1 inhibited. The release of oxidized DNA from the mitochondria and detection in the cytosol is NLRP3-dependent^14^. Herein, we show that *NLRP3*^*−/*−^ or NLRP3-drug inhibited states, which block the release of mitochondrial DNA, can still lead to oxidation and release of DNA that is of nuclear origin (Fig. 4a–c). Oxidation of either mitochondrial or nuclear DNA released to the cytoplasm can act as a DAMP. Both forms of oxidized DNA that elicit an inflammation response in this way are linked to aging and age-related pathologies, so-called “inflammaging”^48,49^. We demonstrate that NLRP3 inhibition, whether by genetic deletion or pharmacological inhibition, leads to cGAS-STING compensation and IFN-β production. We propose a general causal mechanism in that inhibiting OGG1-mediated DNA repair causes an increase in cytosolic oxDNA. This increase in cytosolic oxDNA would normally activate the NLRP3 inflammasome, but TH5487 and SU0268 prevent NLRP3 from interacting with oxDNA and inflammasome assembly. As we are inhibiting both nuclear and mitochondrial OGG1, an initial cytosolic increase in oxDNA could arise from either source^11^. Our results show an increase in cytosolic oxDNA, which has been shown herein to activate cGAS-STING and increase IFN-β^50^. Yet, we observe a decrease in cytosolic D-loop mtDNA when NLRP3 is inhibited (Fig. 4b, c). The total mtDNA decrease suggests mitophagy has occurred, which is known to be associated with cGAS activation^51^. In this case, an initial efflux of ox-mtDNA would activate cGAS-STING and cause mitophagy.

The interplay between IFN-β and IL-1β is complex, as these pathways frequently cross-regulate one another in an antagonistic manner^52,53^. IFN-β can inhibit inflammasome activity and subsequently reduce IL-1β production by activating signaling cascades that counter inflammasome activation^17,53^. Conversely, IL-1β can suppress IFN-β responses under certain inflammatory conditions, creating an environment that dampens type I interferon signaling^54^.

Chronic inflammation involving IL-1β and the NLRP3 inflammasome has been implicated in various age-related diseases, including Alzheimer’s disease (AD)^55–58^. Although our study does not focus on AD directly, AD serves as a well-studied example of how persistent inflammation, or “inflammaging”, can contribute to disease progression^59–61^.

Studies using NLRP3 knockout models in animals indicate that NLRP3 deficiency reduces neuroinflammation and delays cognitive decline in AD models, underscoring NLRP3’s role in AD pathogenesis^62^. Elevated IL-1β levels in post-mortem AD brain tissues, along with correlations between cerebrospinal IL-1β and cognitive decline, further highlight the relevance of this pathway in human AD^63,64^. Additionally, interactions between Aβ and NLRP3 in microglia create a pro-inflammatory feedback loop, perpetuating neuronal damage and exacerbating disease progression^65^. However, OGG1 is diminished in AD brains, and OGG1-mediated repair appears more protective in this context^66^. Thus, AD represents a disease where stimulation of OGG1 activity rather than inhibition might be beneficial. In this setting, enhancing OxDNA repair while simultaneously inhibiting NLRP3 inflammasome with TH10785 could be a therapeutic strategy. In contrast, in other strong inflammatory diseases, it may be more desirable to target OGG1-mediated recruitment of NF-kB with inhibitors like TH5487, which can increase pro-inflammatory cytokines, including Cxcl2 and TNF-α, known to be key mediators in gout, rheumatoid arthritis, asthma and sepsis^17,67^. These insights suggest that targeting the NLRP3 inflammasome to reduce IL-1β release and neuroinflammation could potentially delay the onset or progression of AD and other age-related diseases associated with chronic inflammation^57,58,68,69^. Selective targeting of the NF-kB induced priming and NLRP3 activation may play critical roles in the treatment of inflammaging.

But strictly inhibiting IL-1β is not the only inflammaging remedy. Loss of normal intercellular ion concentrations during pathogen or toxicant exposure and tissue damage can lead to the destabilization of mitochondrial and nuclear DNA sources^70–72^. The ability to inhibit or promote the priming aspect of inflammasome activation while simultaneously inhibiting or promoting repair can have a significant impact on inflammaging. The logic is as follows: during mtDNA replication or repair, G-rich regions are especially susceptible to oxidation and DNA strand breaks, which can cause deletions^73^. The mitochondrial theory of aging suggests that insufficient availability of peptide synthesis will decrease electron transfer across ETC respiratory complexes^74^, which stalls, leading to early transfer of electrons to O_2_^75^. The cycle of deletions^76^ and mutations^77^, which are uncommon before the age of 40, exponentially accumulates during old age. The accumulation of oxidative damage to mtDNA and nuclear DNA is an important mechanism of aging^78,79^. The mitochondria can control cell fate via efflux of DNA from mPTP (mitochondrial Permeability Transition Pores) to the cytosol^11,80,81^. We find herein that the release of mtDNA from the mitochondria is NLRP3-dependent. Inhibiting this process stops production of IL-1β, but leads to an increase from nuclear sources. The NLRP3 inhibitor herein, TH10785, which simultaneously activates hOGG1, may facilitate repair of nuclear DNA, leading to a reduction in TLR9-induced responses due to the presence of cytosolic nuclear DNA. Given that attempts to repair DNA during high ROS can lead to deletions and mutations, there is a desire to inhibit or promote DNA repair depending on cytosolic DNA levels arising from the nucleus.

Moreover, alcohol abuse can also have effects on mtDNA stability^82–84^. A single dose of ethanol causes extensive degradation and depletion of hepatic mtDNA within 2 h in mice^85,86^. After 4 days of alcohol administration, numerous deletions of mtDNA prevent mitochondrial replication because there are not enough intact copies of templates. This leads to mtDNA depletion for several days until mtDNA is restored once alcohol intake stops in white blood cells, which have a quick turnover^87^. Excessive alcohol and fructose have a profound impact on liver mitochondrial dysfunction and NLRP3 expression, which ultimately progress to metabolic dysfunction-associated steatohepatitis (MASH) and hepatocellular carcinoma (HCC)^88^. Subsequent experiments will evaluate the contribution of NLRP3 to these diseases using the small-molecule inhibitors illustrated herein.

The ability to simultaneously target the priming and activation phases of inflammasome activation may represent a promising mechanistic advantage. Future experiments will involve optimizing the specificity for biphasic OGG1/NLRP3 targeting in vivo as well as defining molecular determinants of NLRP3 recognition for oxidized DNA and drugs described^89,90^.

## Methods

Further information can be found in Supplemental Methods

### Sex as a biological variable

Our study examined male and female animals, and similar findings are reported for both sexes.

### Primary human PBMC inflammasome activation in the presence of TH5487, SU0268, TH10785, and MCC950

PBMCs from healthy individuals (OrganaBio) were seeded at 2 × 10^6^/mL in 96-well plates (200 µL/well) in Opti-MEM Reduced-Serum Media (Gibco). Cells were primed with 1.6 µg/mL LPS (Thermo Fisher) for 3 h. During the third hour, cells were treated with 0.1–100 µM TH5487 (Selleck Chemicals), SU0268 (MedChemExpress), TH10785 (MedChemExpress), MCC950 (Sigma), or vehicle for 1 h. After the 1 h drug treatment, the LPS-only samples were harvested, and 20 µM nigericin (Sigma) was added to the remaining samples for 45 min. Cells were then harvested by spinning the plates at 1000 x *g* for 5 minutes. The supernatant was removed and saved for IL-1β western blots and ELISAs, and the cells were washed 3 times with sterile pre-chilled PBS, lysed with 50 µL of RIPA buffer (Boston BioProducts) supplemented with an EDTA-free protease/phosphatase inhibitor cocktail (Roche) by rotating for 5 minutes at 4 °C. The lysed cells were spun at 14,000 × *g* for 15 min. Next, 40 µL of the clarified lysate was removed and saved for analysis. The protein concentration of each fraction (supernatant and lysate) was evaluated using a Bradford Assay (BioRad) for western blot and qPCR analysis.

### FCAS PBMC inflammasome activation in the presence of TH5487 and MCC950

PBMCs from patients harboring the L353P FCAS mutation were a generous gift from Dr. Hal Hoffman (UCSD). Cells were seeded at 2 × 10^6^/ml in 96-well plates in Opti-MEM Reduced-Serum Media (Gibco). Cells were primed with 1.6 µg/ml LPS (Thermo Fisher) for 3 h. During the third hour, cells were treated with 0.1–100 µM TH5487 (Selleck Chemicals), MCC950 (Sigma), or vehicle for 1 h. After the 1 h drug treatment, all the cells were harvested by spinning the plates at 1000 × *g* for 5 min. The supernatant was removed and saved for IL-1β western blots and ELISAs, and the cells were washed 3 times with sterile pre-chilled PBS, then the pellet was lysed as described above for western blot and qPCR analysis

### Bone marrow-derived mouse macrophage cell culture and inflammasome activation assay from WT and NLRP3^LP351P/+Esr1cre^ mice

Bone marrow cells were flushed using DMEM supplemented with 10% FBS, 1% PSG, 1% NaPyruvate and 100 ng/mL M-CSF, and cultured overnight in tissue culture flasks at 37 °C and 5% CO₂ to allow fibroblast adherence. The following day, non-adherent cells were transferred to Petri dishes and differentiated in medium containing 100 ng/mL M-CSF. On day 4, adherent macrophages were washed with cold PBS, detached using pre-warmed 2 mM EDTA in HBSS, centrifuged (400 × *g*, 5 min, 4 °C), and were seeded at 5 × 10^4^ cells per well in 96-well plates with 20 ng/mL M-CSF and incubated overnight prior to 24 h treatment with 4-hydroxytamoxifen. Cells were subsequently primed with 20 ng/mL GM-CSF overnight, followed by stimulation with ultrapure LPS (InvivoGen) for 4 h. MCC950 was added 1 h prior to LPS; TH5487 and SU0268 were added 3 h post-LPS; and ATP (Sigma-Aldrich) was added to WT cells during the final 30 min of stimulation. Supernatants were collected at 4 h, and IL-1β concentrations were quantified using ELISA (R&D Systems, DY401).

### THP1 NLRP3 inflammasome activation by Antimycin A in the presence of TH5487 and SU0268

The viability and concentration of a culture of immortalized human THP1 cells were checked as described above. On day 0, cells at viability >95% and 0.5 × 10^6^ cells/mL were split into 6-well TC-treated plates (Corning) with 2 mL of cells per well and allowed to double overnight. On day 1, 500× lipopolysaccharide (Thermo Fisher) was added to each well for 16 h for a final concentration of 500 ng/mL. On day 2, drugs TH5487 (Selleck Chemicals) or SU0268 (MedChemExpress) were serially diluted in DMSO (Fisher Scientific) such that the addition of any concentration of inhibitor was 1% of the final volume of cells. The inhibitors were added at concentrations ranging from 0.1–100 µM for 1 h, along with a vehicle control added to the 0 µM conditions LPS-only wells were harvested, and Antimycin A (ThermoFisher) was resuspended in DMSO and added to each well for a final concentration of 10 µM for two hours. The supernatant and cells were separated by spinning at 300 x *g* for 5 min at 4 °C. The supernatant fraction was removed from the cell pellet and clarified by spinning at 3000 x *g* for 5 min. The cell pellet was resuspended in 1 mL ice-cold PBS and re-pelleted by spinning at 1000 × *g* for 5 min at 4 °C. The PBS was removed, and the pellet was further processed to separate the cytosolic, mitochondrial, and whole cell fractions. One-third of the pellet was used for whole-cell lysis as described above. The rest of the pellet was resuspended in mitochondrial extraction buffer (220 mM mannitol, 70 mM Sucrose, 20 mM HEPES KOH pH 7.5, 1 mM EDTA, 2 mg/mL BSA) and passed through a 25-G syringe 20 times on ice. Samples were centrifuged at 1000 × *g* for 15 min at 4 °C, and the supernatant was saved in a new tube. These tubes were further centrifuged at 10,000 × *g* for 10 min at 4 °C to collect the mitochondria. The supernatant was saved as the cytosolic fraction, and the pelleted mitochondrial fraction was lysed using the whole cell lysis method. The protein concentration of each fraction (supernatant, whole cell, cytosolic, and mitochondrial) was evaluated using a Bradford Assay (BioRad) and western blots were run to quantify protein expression.

### Co-immunoprecipitation of NLRP3 inflammasome components

Immortalized human THP1 cells were activated and treated with various small molecules as described above. Cells were lysed gently using 300 µL ice-cold 25 mM Tris pH 7.4, 0.15 M NaCl, 0.001 M EDTA, 1% NP40, 5% glycerol, and protease/phosphatase inhibitor and rotating at 4°C for 5 min. The lysate was clarified by spinning at 13,000 × *g* for 10 min and the supernatant was saved for protein concentration determination using Bradford assay (abcam ab102535) and co-immunoprecipitation. 100 µg of each sample was used for analysis, with the total volume normalized to 100 µL. An NLRP3 capture antibody (ABclonal A12694) was added to each sample at a 1:200 ratio. The samples were mixed at room temperature for 1 h. While mixing, 50 µL of protein G beads (Millipore Sigma LSKMAGG10) per sample were washed three times with 500 µL PBS + 0.01% Tween using a DynaMag-2 magnet. The beads were resuspended in PBS + 0.01% Tween such that an equal amount of bead suspension (100 µL) could be added to the antibody/sample mixture. This new mixture was rotated at room temperature for 1 h. After incubating, samples were pulled back onto the magnet, the supernatant/unbound fraction was removed, and the beads were washed three times again with 500 µL PBS + 0.01% Tween. Samples were eluted off the beads by adding 15 µL of 4× sample buffer (NuPage) + 6 µL of 10× Reducing agent (NuPAGE) + 39 µL DEPC water and heating for 5 min at 90 °C. The boiled samples were placed on a magnet to pull back the beads. The supernatant was loaded onto a NuPAGE™ 4 to 12%, Bis-Tris 1 mm 15-well mini-gels at 200 V for 30 min. Samples were transferred to PVDF membranes, blocked with 2.5% BSA in TBST, and probed with a primary antibody against NLRP3 (Adipogen), NEK7 (abcam), ASC (Adipogen), or pro-caspase-1 (Cell Signaling). Blots were incubated with an HRP-linked secondary antibody (either mouse, rabbit, or goat, depending on the species of the primary) and imaged using the iBright 1500 Imaging system. The intensities of the bands were quantified using the iBright Analysis Software, where the amount of each protein that co-immunoprecipitated with NLRP3 was quantified by comparing the intensity of the bands associated with pro-caspase-1, NEK7, and ASC to the corresponding NLRP3 band. The intensity values were plotted and analyzed using GraphPad Prism and a one-way ANOVA.

### Immunofluorescence assays

Immortalized mouse BMDMs were plated into 12-well plates containing poly-l-lysine (NC9663893; Fisher Scientific) coated coverslips and left to adhere overnight. Cells were treated as previously described in the methods. After cell treatments, cells were washed with phosphate-buffered saline (PBS) to remove medium before cells were fixed with 4% paraformaldehyde (PFA) (sc-281692; Santa Cruz Technology) for 30 min. Coverslips were washed with PBS again and permeabilized with 0.2% Triton X-100 for 10 min while on ice. After blocking with 0.5% bovine serum albumin (BSA) blocking buffer for 30 min, coverslips were incubated with primary antibodies (diluted in 0.5% BSA blocking buffer) overnight at 4 °C. Primary antibodies used were for NLRP3 (AdipoGen, Cryo2 AG-20B-0014-C100) and ASC (AdipoGen AL177). The following day, coverslips were washed before being incubated with secondary antibodies (1:5000 dilution in 0.5% BSA blocking buffer) for 40 min at room temperature. Finally, coverslips were mounted onto glass slides with ProLong Gold antifade reagent with 4′,6-diamidino-2-phenylindole (DAPI) (P36931; Invitrogen) to stain cell nuclei. Images were acquired on a Zeiss LSM-900 microscope with Airyscan using ×20 magnification. The excitation lasers used to capture the images were 488, 568, 630, and 405 nm using Alexa 488–, Alexa 568–, and Alexa 647–conjugated secondary antibodies. The same brightness/contrast profile was applied to all images within the same experiment. Five to ten images were captured per condition, where each image was considered a field. Zeiss and ImageJ imaging software were used for image analyses (an average of 10 cells per field, depending on magnification and cell type). Roughly 1000–1800 total cells were quantified per condition. We performed a Pearson’s Correlation Coefficient analysis and a Manders’ Coefficient analysis with the JACoP Plugin using FIJI software. Our manual percentage ASC speck count of these images validated the automated quantification.

### Isolation and quantification of iBMDM and primary human PBMC RNA by reverse transcription PCR (RT-PCR) and Real-Time PCR (qPCR)

Immortalized iBMDM cells or primary human PBMCs were treated in the presence of OGG1 small molecules as described above. Cells were washed with pre-chilled PBS and harvested at 1000 × *g* for 5 min. The total RNA was isolated from the cells using the RNeasy Mini RNA Purification kit (Qiagen) per the manufacturer’s instructions. The RNA concentration was checked via A260 absorbance, and 5 µg of each sample was used in the reverse transcription reaction using the iScript Advanced cDNA Synthesis Kit (Bio-Rad) per the manufacturer’s instructions. Real-time PCR reactions were prepared with primers against *nlrp3*, and *asc*, along with *gapdh* as a control. Reactions were run using the SsoAdvanced Universal SYBR® Green Supermix (Bio-Rad). The qPCR reactions were run on the CFX Duet Real-Time PCR machine using the following conditions: Initial denaturation at 98 °C for 3 min, then 40 cycles of denaturation at 98 °C for 15 s and annealing at 60 °C for 30 s. This was followed by a melt curve from 65 °C to 95 °C with 0.5 °C steps and 5 s per step. The readout was visualized using the BioRad CFX Maestro Software. The relative amounts of mRNA of either NLRP3 or ASC were calculated using GAPDH as a control. The final ΔΔCq values were plotted and analyzed using GraphPad Prism and a one-way ANOVA.

### Isolation of THP1 and PBMC cytosolic, mitochondrial, and nuclear DNA

Wild-type human THP1s or primary human PBMCs were treated in the presence or absence of hOGG1 small molecules as described above. Cells were then washed with pre-chilled PBS and harvested at 1000 × *g* for 5 min. To isolate the nuclear fraction, the pellet was resuspended in a hypotonic lysis buffer (HLB) (10 mM Tris HCl, pH 7.5, 10 mM NaCl, 3 mM MgCl_2_, 0.3% NP-40, 10% glycerol) and incubated rotating at 4 °C for 10 min. The mixture was then centrifuged for 2 min at 200 x *g*. The supernatant was saved as the cytosolic fraction in new tubes, the remaining nuclear pellet was resuspended in HLB, and centrifuged again for 2 min at 200 x *g* to wash. This step was repeated two more times. The washed pellet was then resuspended in nuclear lysis buffer (NLB) (20 mM Tris HCL, pH 7.5, 150 mM KCL, 3 mM MgCl_2_, 0.3% NP-40, 10% glycerol), vortexed, and lysed with an 18-gauge needle syringe by pulling up and expelling the liquid 10 times on ice. Both the tube with the pre-saved cytosolic and the newly lysed nuclear fraction were centrifuged for 15 min at 20,000 × *g*. The supernatant from each was collected into new tubes and used for subsequent DNA purification.

To isolate the mitochondrial fraction, the pellet was resuspended in cold mitochondrial extraction buffer (22 mM mannitol, 70 mM sucrose, 20 mM Tris Base pH 7.5, 1 mM EDTA, 2 mg/mL BSA, and 1 tablet of protease and phosphatase inhibitor (Roche)), then passed through a 25-G syringe (Fisher #14-817-133) 20 times on ice. The mixture was spun at 1000 × *g* for 15 min at 4 °C, and the pellet was discarded. A second spin on the supernatant was performed at 10,000 × *g* for 10 min at 4 °C. The supernatant was saved for further purification as the cytosolic fraction, and the pellet was saved for further purification of the mitochondrial fraction.

### Real-time PCR (qPCR) analysis of cytosolic, mitochondrial, and nuclear DNA

The cytosolic, mitochondrial, and nuclear fractions of DNA were purified using the AllPrep DNA/RNA Mini Kit (Qiagen) per the manufacturer’s instructions. After purification was completed, the DNA concentration was evaluated by reading the A260 on a nanodrop. qPCR reactions were set up with primers for *hTert* (nuclear DNA), *D-loop* (mitochondrial DNA), and *GAPDH* (control) using the SsoAdvanced Universal SYBR Green Supermix (BioRad # 1725270) per the manufacturer’s instructions. The qPCR reactions were run on the CFX Duet Real-Time PCR machine using the following conditions: Initial denaturation at 98 °C for 3 min, then 40 cycles of denaturation at 98 °C for 15 s and annealing at 60 °C for 30 s. This was followed by a melt curve from 65 °C to 95 °C with 0.5 °C steps and 5 s per step. The readout was visualized using the BioRad CFX Maestro Software. The Cq values obtained for GAPDH DNA abundance served as normalization controls for the DNA values obtained from the test genes. The final ΔΔCq from the cytosolic, mitochondrial, and nuclear fractions of *hTert* and *D-loop* were compared, plotted, and analyzed using GraphPad Prism and a one-way ANOVA.

### Streptavidin-affinity grid preparation and NLRP3 complex visualization using cryoEM

NLRP3:TH5487 Decemer was mixed with and biotinylated non-oxDNA and incubated on ice for 1 h. The prepared grids are not glow-discharged. A total of 4 µL of sample was added to the non-glow-discharged grids containing a lipid-biotin layer. The grids were placed in the humidity chamber to make them hydrophilic for 5–10 min. The grids were then inverted and touched to wash buffer (50 mM Tris, 150 mM NaCl,10 mM MgCl_2_, 5% glycerol, 0.01 mM Th5487, 1 mM ADP, pH 7.5) 1–3 times. After touching the grid to buffer, the grid was then touched to 0.01% beta-octyl-glucopyranoside (BOG) in wash buffer. Then, the grid was released in a 20 µl 0.01% BOG drop, picked up with plunging tweezers, inserted into a Leica plunger, and blotted once only on the side with the sample. An additional 4 µL 0.01% BOG was added to the top of the grid. The sample was blotted again, plunged, clipped and loaded onto a Titan Krios microscope equipped with a Falcon 4i camera. A total of 26,390 movies were collected at 0.743 Å /pix at a total dose of 40 e/Å^2^, an exposure time of 3.25 s, and 37 frames per movie across a defocus range of −1.5 to −2.5 µm. After collection, the movies were motion corrected using RELION MotionCore2, and the streptavidin lattice was subtracted from all micrographs using MatLab^43^. Corrected micrographs and FFT were visualized using EMAN2^91^.

### Immortalized human THP1 cell culture

Immortalized wild-type THP1 cells were purchased from InvivoGen (THP1-Null). Frozen vials were thawed in a water bath and then resuspended in 50 mL of culture media (RPMI no phenol red (Gibco), 10% heat-inactivated FBS (Sigma), 1× Penicillin-Streptomycin-Glutamine (Gibco), 1× non-essential amino acids (Gibco), 1× Sodium Pyruvate (Gibco)). The cells were spun down at 300 × *g* for 5 min at 4 °C, and the pellet was resuspended in 5 mL of culture media. The cells were counted using a CountesS4 (Invitrogen) and resuspended at 0.3 × 10^6^ cells/mL, doubling every 24–48 h, and maintained according to the manufacturer’s instructions. The cells were counted by diluting 10 µL of cells with 10 µL of Trypan Blue Stain (Thermo Fisher), loading that dilution onto a CountessTM Cell Counting Chamber Slide (Thermo Fisher), and evaluating using a CountessTM 3 FL Automated Cell Counter (Thermo Fisher).

### THP1 inflammasome activation in the presence of TH5487, SU0268, and TH10785

The viability and concentration of a culture of immortalized human THP1 cells were checked as described above. On day 0, cells at viability >95% and 0.5 × 10^6^ cells/mL were split into 6-well TC-treated plates (Corning) with 2 mL of cells per well and allowed to double overnight. On day 1, 500× lipopolysaccharide (Thermo Fisher) was added to each well for 16 h for a final concentration of 500 ng/mL. On day 2, LPS-only wells were harvested, and drugs TH5487 (Selleck Chemicals), SU0268 (MedChemExpress), TH10785, or OLT1177 (MedChemExpress) were serially diluted in DMSO (Fisher Scientific) such that the addition of any concentration of inhibitor was 1% of the final volume of cells, along with a vehicle control added to the 0 µM conditions. Then the inhibitors were added at concentrations ranging from 0.001 to 100 µM for 1 h. Next, LPS-only wells were harvested. To activate NLRP3, 20 µM nigericin (Sigma), 20 µM imiquimod (MedChemExpress) or 4 mM ATP was added to each well for 1 h. To activate AIM2, cells were transfected with snon-ox-mtDNA in the presence of Lipofectamine 2000 (ThermoFisher). Cells and supernatant fractions could then be isolated for viability and western blot analysis. The supernatant and cells were separated by spinning at 300 × *g* for 5 minutes at 4 °C. The supernatant fraction was removed from the cell pellet and clarified by spinning at 3000 × *g* for 5 min. The cell pellet was resuspended in 1 mL ice-cold PBS and re-pelleted by spinning at 1000 × *g* for 5 min at 4 °C. The PBS was removed from the pellet, and the cells were lysed with 300 µL of RIPA buffer (Boston BioProducts) supplemented with an EDTA-free protease/phosphatase inhibitor cocktail (Roche) by rotating for 5 min at 4 °C. The lysed cells were spun at 14,000 × *g* for 15 min. Two hundred microliter of the clarified lysate was removed and saved for analysis. The protein concentration of each fraction (supernatant and whole cell) was evaluated using a Bradford Assay (BioRad), and western blots were run to quantify protein expression.

### Immortalized bone marrow-derived mouse macrophage cell culture

Immortalized wild-type and NLRP3-knockout bone marrow-derived mouse macrophages were generously provided to us by Michael Karin at the University of California, San Diego. A frozen vial of cells at 1 × 10^7^ cells/mL was thawed in a water bath and then resuspended in 50 mL of culture media DMEM (Thermo Fisher) supplemented with 10% heat-inactivated FBS (Sigma) and 1% Penicillin-Streptomycin (Thermo Fisher). The cells were spun down at 300 × *g* for 5 min at 4 °C, and the pellet was resuspended in 5 mL of culture media. The cells were counted as described above. Cells typically doubled in 24–48 h, where they were then scraped from the bottom of the plate using Bio-One Cell Scrapers (Fisher Scientific) and then spun down, counted, and expanded.

### Macrophage NLRP3 inflammasome activation in the presence of TH5487, SU0268, and TH10785

The viability and concentration of a culture of immortalized mouse macrophages were checked as described above. Cells at viability >95% and 0.5 × 10^6^ cells/mL were split into 6-well TC-treated plates (Corning) with 2 mL of cells per well and allowed to adhere and double overnight. The next day, 500× lipopolysaccharide (Thermo Fisher) was added to each well for 1 h for a final concentration of 500 ng/µL. Next, drugs TH5487 (Selleck Chemicals), SU0268 (MedChemExpress), or TH10785 were serially diluted in DMSO (Fisher Scientific) such that the addition of any concentration of inhibitor was 1% of the final volume of cells. The inhibitors were added at concentrations ranging from 0.1–100 µM for 1 h, along with a vehicle control added to the 0 µM conditions. Next, LPS-only wells were harvested and 20 µM nigericin (Sigma) or 4 mM ATP was added to each well for 1 h. Cells and supernatant fractions could then be isolated for viability and western blot analysis. To collect samples for western blot analysis, the supernatant was removed from each well and clarified by spinning at 3000 × *g* for 5 min. The cells left on the plate were washed with ice-cold PBS and then lysed with 300 µL of RIPA buffer (Boston BioProducts) supplemented with an EDTA-free protease/phosphatase inhibitor cocktail (Roche). The lysis took place for 5 min, rocking at 4 °C. The lysed cells were then collected into 1.5 mL tubes and spun at 14,000 × *g* for 15 min. Next, 200 µL of the clarified lysate was removed and saved for western blot analysis. The protein concentration of each fraction (supernatant and whole cell) was evaluated using a Bradford Assay (BioRad), and western blots were run to quantify protein expression.

### IL-1β secretion quantification by ELISA

Inflammasome activation was measured by an enzyme-linked immunosorbent assay (ELISA) against human or mouse IL-1β, depending on whether the samples were from primary human PBMCs, THP1 cells, or iBMDMs. Both human and mouse IL-1β kits were purchased from Abcam and used according to the manufacturer’s instructions. Each assay was performed the same, with specific reagents for each kit. Briefly, Standards were reconstituted in Standard Diluent Buffer and serially diluted 5 times from 500–15.6 pg/mL (human) or 6 times from 100–1.56 pg/mL (mouse). A buffer alone blank control was also included for both. Included antibody-coated microplate strips were removed, and 50–100 µL of each standard, blank, and sample was added to the appropriate cells. Antibody was added to the wells for 1 h (mouse) or 3 h (human). Sample wells were washed with the included wash buffer 3 times. Specifically for the human ELISA, a secondary streptavidin-HRP antibody was added to the wells for 30 min, and then washed. TMB solution was added to each well of both ELISAs for 10 min shaking at 400 rpm in the dark. Stop solution was added to each well, and the absorbance at 450 nm was read immediately using a Synergy H1 plate reader (BioTek). The data were plotted and analyzed using GraphPad Prism and a one-way ANOVA.

### Protein quantification and analysis by Western Blot

Total protein concentration was determined and normalized using a Bradford Assay (abcam #ab102535). To evaluate proteins secreted from the cells (Caspase-1 and IL-1β), the supernatant fractions were run on Western blots. To evaluate proteins expressed inside the cell (NLRP3, Caspase-1, FEN1, hOGG1, and Actin), the lysate fraction was run. Samples were diluted with LDS sample loading buffer and reducing agent (Invitrogen), each at a final concentration of 1×. The samples were boiled at 90 °C for 5 min and run on NuPAGE™ 4 to 12%, Bis-Tris 1 mm 15-well mini-gels at 200 V for 30 min at 4 °C. Samples were transferred to PVDF membranes at 4 °C using an Invitrogen mini blot module for 1 h at 25 volts. Membranes were then blocked with 2.5% BSA (Geminibio) in TBST, and probed with a primary antibody against the specific protein diluted to the manufacturer’s recommendation in 2.5% BSA in TBST. Blots were incubated with an HRP-linked secondary antibody (either mouse, rabbit, or goat, depending on the species of the primary) and imaged using the iBright 1500 Imaging system (Thermo Fisher). Western blot band intensities were quantified using iBright image analysis software. For each blot, a region of interest (ROI) was drawn around each band, and an identically sized ROI was placed in a nearby area lacking signal to determine local background. Background intensity was subtracted from the raw band intensity to obtain background-corrected values. The background-corrected intensity of the positive control band (LPS/ATP or LPS/nigericin) was measured for each blot and defined as 100%. To normalize all other samples relative to this control, the background-corrected intensity of each sample was divided by the intensity of the corresponding positive control and multiplied by 100, yielding values expressed as a percentage of the control. When applicable, the target protein signal was further normalized to the loading control (β-actin or GAPDH, or total protein) by dividing the background-corrected target band intensity by the background-corrected loading control intensity for the same lane prior to normalization to the positive control. Quantification was performed on independent biological replicates, and normalized values were used for downstream statistical analysis using GraphPad Prism

### Ponceau staining

To verify consistent loading in the supernatant samples, Ponceau staining was performed per the manufacturer’s instructions (Sigma, #P7170). After western blot analysis as described above, membranes were incubated with 30 mL of Ponceau S solution for at least 15 min while rocking. To destain the membrane, the Ponceau S solution was removed, and the membrane was incubated with 30 mL of DI H2O for 1 min, then imaged using the iBright 1500 Imaging system (Thermo Fisher).

### Cell death quantification by LDH measurement

To quantify the viability of cells pre/post-treatment, the amount of lactate dehydrogenase (LDH) secreted into the media was measured. This was done using the CytoTox 96 Non-Radioactive Cytotoxicity Assay (Promega) per the manufacturer’s instructions^92^. A 50 µL aliquot of all cell samples and a no-cell control were added to a 96-well plate and incubated with 50 µL CyTox 96 Reagent in the dark for 30 min at room temperature. After the incubation, 50 µL of Stop Solution was added to each well, and the absorbance was read at 490 nm using a Synergy H1 plate reader (BioTek). The percentage of toxicity was calculated based on the Maximum LDH release controls and plotted and analyzed using GraphPad Prism and a one-way ANOVA.

### Purification of full-length NLRP3 decamer bound to TH5487

Wild-type NLRP3 was cloned into the mammalian expression vector pcDNA3.1HisB. The plasmid was expressed in DH5α cells (New England Biolabs) and purified using the PureLink HiPure Plasmid Maxiprep Kit (Thermo Fisher). The protein was expressed using the Expi293 Expression System (Thermo Fisher) in the presence of 0.01 mM TH5487 in DMSO. Cells were grown in Expi293 expression media until they reached a concentration of 3 × 10^6^ cells per milliliter and sustained viability of ≥95% live cells. At that time, 1 μg of expression vector was transfected per every 1 mL of cells with Expifectamine reagent. Once the cells reached viability of ≤80% live cells, they were harvested by spinning at 300 rpm for 5 min. The supernatant/dead cells were aspirated from the top, and the pellet was washed with cold PBS. Cells were resuspended in lysis buffer containing 50 mM Tris, 150 mM NaCl, 0.5 mM TCEP, 10 mM MgCl_2_, 0.01 mM TH5487, 1 mM ADP, 0.1 mM PMSF pH 7.5. The suspension was lysed by sonication with 5 s on, 10 s off, for a total of 4 min at 40%. The soluble lysate fraction was isolated by centrifuging at 100,000 × *g* for 1 h and further clarified by filtering through a 0.45 µm syringe filter. A HisTrap FF crude 5 mL column was equilibrated in binding buffer (50 mM Tris, 150 mM NaCl, 10 mM MgCl_2_, 5% glycerol, 0.01 mM Th5487, 1 mM ADP, pH 7.5). After the sample was loaded onto the column, it was washed with 10 column volumes (CV) of the wash buffer above, then eluted with 250 mM imidazole using a 50% gradient of the buffer 50 mM Tris, 150 mM NaCl, 10 mM MgCl_2_, 5% glycerol, 500 mM imidazole, 0.01 mM TH5487, 1 mM ADP, pH 7.5. Peak fractions were pooled, and the affinity-purified protein was crosslinked using 0.5 mM bis(sulfosuccinimidyl)-suberate (BS3) for 30 minutes at 4 °C. BS3 is a short, amine-reactive cross-linker used primarily stabilize pre-existing interactions. This cross-linking was used as a mild stabilization strategy to capture structurally relevant assemblies in the presence of the drug. The reaction was quenched by the addition of 100 mM ammonium hydrogen carbonate for 15 min at 4 °C. The cross-linked protein was further purified by size exclusion using a HiLoad 16/600 Superose 6 pg size exclusion column equilibrated in binding buffer. Peak fractions were concentrated to ~10 mg/mL and visualized by SDS and Native gels, and western blots against NLRP3.

### AlphaFold structural modeling of NLRP3 bound to DNA

AlphaFold simulations were performed using the AlphaFold3 Google server on the NLRP3 sequence alone in combination with the DNA template. For these simulations, we used the pyrin domain of NLRP3 (residues 1–85) or a full monomer (residues 1–1036) from PDB 7PZC (PMID: 35114687). The DNA sequence used was the DNA sequence published in PDB 1EBM (PMID: 10706276), which is the oxDNA used in the structure of hOGG1 bound to DNA. Predicted complexes were evaluated using AlphaFold confidence metrics, including per-residue pLDDT and predicted aligned error (PAE), and the highest confidence models were selected. Structural visualization and figure preparation were performed using ChimeraX.

### NLRP3 bound to TH10785 model generation using SWISS-MODEL

The amino acid sequence of hOGG1 that aligned with the NLRP3 pyrin domain (249-325) was saved as a “.pdb” file from the published structure of hOGG1 bound to ox-DNA (PDBID: 1EBM)^14^. The sequence was uploaded to SWISS-MODEL as the User Template Modeling template file^93^. Then, various truncations of the NLRP3 pyrin domain were loaded as test target files to see which generated a viable model. Empirically, NLRP3 amino acids 1–85 were chosen. The SWISS-MODEL projection produced one model of NLRP3 based on the template hOGG1 structure. This model was further analyzed using ChimeraX to compare it to the published structures of wild-type NLRP3 (PDB1D: 7PZC) and hOGG1 bound to activator TH10785 (PDBID: 7AYY)^94,95^.

### Quantification and statistical analysis

A one-way ANOVA, two-way ANOVA, or nonlinear regression was used to conduct all statistical analyses herein. All statistical analyses were performed as indicated in the figure legends, where N represents the number of replicates. Bar graph data were presented as mean ± SEM. Line graph data were presented as mean ± SD. *P*-values < 0.05 were considered statistically significant.

### Study approval

Studies performed using PBMCs obtained from patients with CAPS and FCAS mice received the approval of the University of California Human Research Protection Program committee (number 180064), and informed consent was obtained from the subjects before the study. All ethical regulations relevant to human research participants were followed.

### Inclusion and ethics

We, the authors, have read the Nature Portfolio Authorship Policy and confirm that this manuscript complies. All authors have contributed to the manuscript and approved the final version.

### Reporting summary

Further information on research design is available in the Nature Portfolio Reporting Summary linked to this article.

## Supplementary information

Supplementary Information

Reporting Summary

## Data Availability

Requests for further information and resources should be directed to and will be fulfilled by the lead contact, Reginald McNulty, 540 Steinhaus Hall, Irvine, CA, 92697, 949-824-5541, rmcnulty@uci.edu. All unique/stable reagents generated in this study are available upon reasonable request. Uncropped western blots are available in supplemental figures 18-24. All numerical data used to generate the data shown are available at the Figshare repository^96^. 10.6084/m9.figshare.30875246. Additional data that support the findings of this study are available from the corresponding author upon reasonable request.

## Code availability

This paper does not report original code.
