# Supplementary material for: TH5487 specifically targets NLRP3 in FCAS patients resistant to MCC950
Source: Commun Biol. 2026 Apr 16;9:528. doi: 10.1038/s42003-026-10008-2 (PMC13086942; doi:10.1038/s42003-026-10008-2)
Supplement: Supplementary file 1 — Supplementary Information [file 42003_2026_10008_MOESM1_ESM.pdf]

SUPPLEMENTAL DOCUMENT

Supplemental Figures and Tables

- Figures S1-S24

- Tables S1-S2

-uncropped gel images for all figures

SUPPLEMENTAL FIGURES AND TABLES

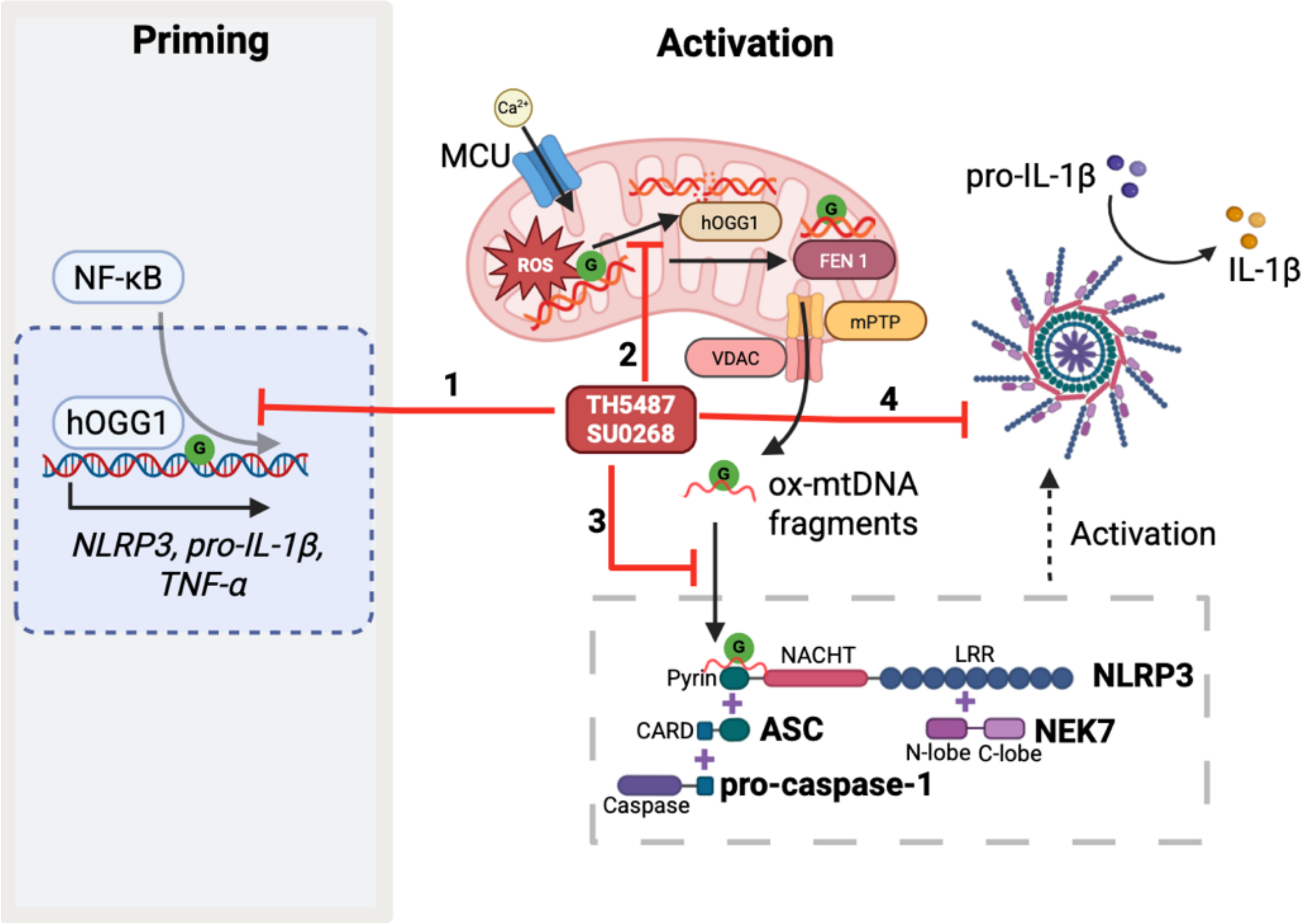

Figure. S1: Repurposed drugs target OGG1 and NLRP3 inflammasome activation. OGG1 inhibitors TH5487 and SU0268 inhibit OGG1 in the nucleus (1) and mitochondria (2), NLRP3's interaction with oxidized DNA (3), and inflammasome activation (4). Inhibition in the nucleus blocks OGG1 from recruiting NF-kB upon sensing ox-DNA (green G). This blocks OGG1-dependent NF-κB-mediated priming, including the upregulation of proteins such as NLRP3, IL-1β, and TNF-α (left, blue dashed box). OGG1 inhibition in the mitochondria results in an increase of mitochondrial ox-DNA, while NLRP3 inhibition blocks the interaction between NLRP3 and ox-DNA, and inhibits inflammasome activation (right).

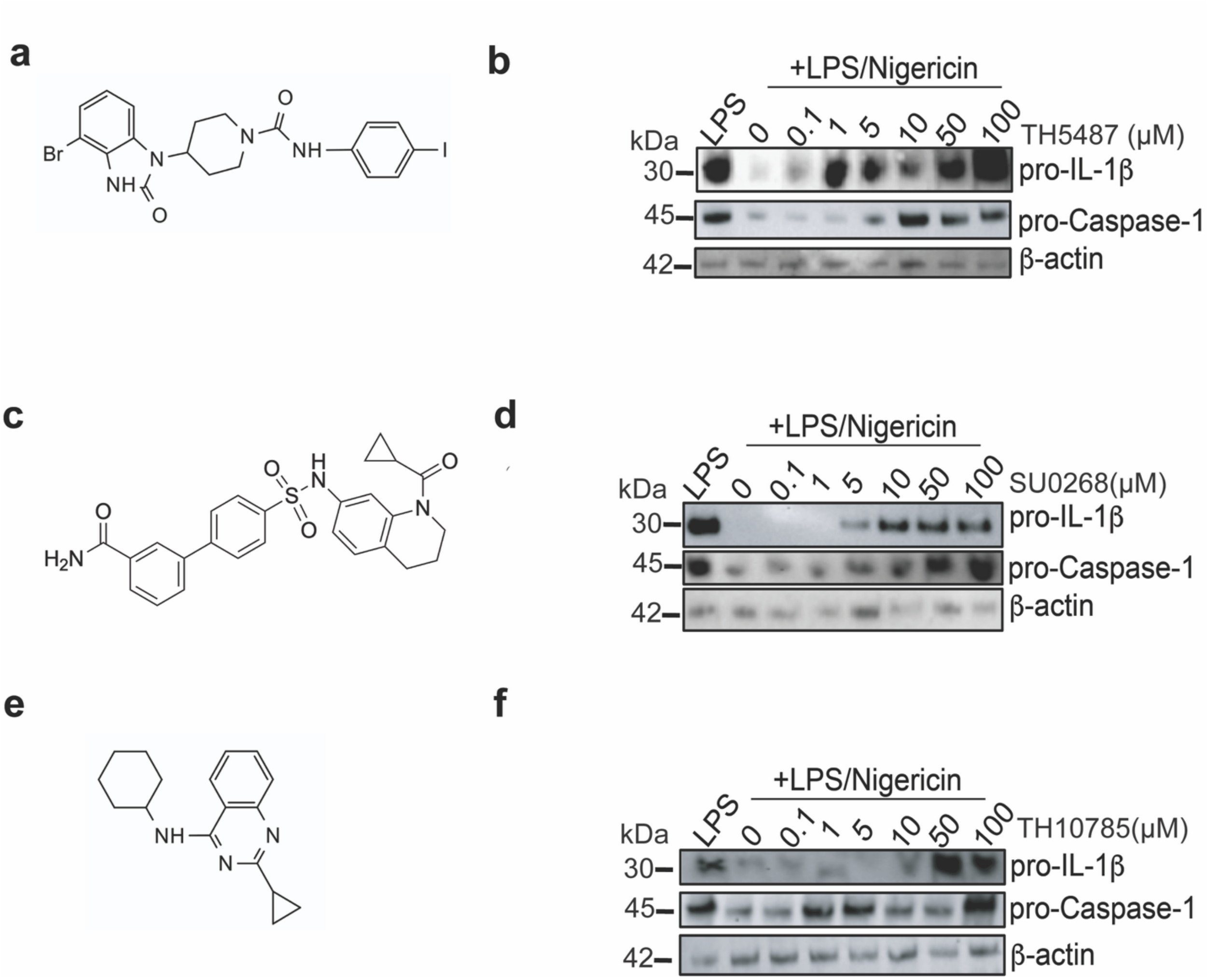

Figure. S2: All three hOGG1 drugs increased the amount of Pro-IL-1β and Pro-Caspase-1 retained inside human PBMC cells, while SU0268 also caused a reduction in priming. A) Chemical structure of hOGG1 inhibitor TH5487 B) Primary human PBMC from healthy donors were primed with LPS, treated with TH5487 at 0.1-100 μM, then activated with nigericin. Cells were lysed, and pro-IL-1β, pro-Caspase-1, and β-actin expression were evaluated by western blot. C) Chemical structure of hOGG1 inhibitor SU0268 D) Primary human PBMC from healthy donors were primed with LPS, treated with SU0268 at 0.1-100 μM, then activated with nigericin. Cells were lysed, and pro-IL-1β, pro-Caspase-1, and β-actin expression were evaluated by western blot. E) Chemical structure of hOGG1 activator TH10785 F) Primary human PBMC from healthy donors were primed with LPS, treated with TH10785 at 0.1-100 μM, then activated with nigericin. Cells were lysed, and pro-IL-1β, pro-Caspase-1, and β-actin expression were evaluated by western blot

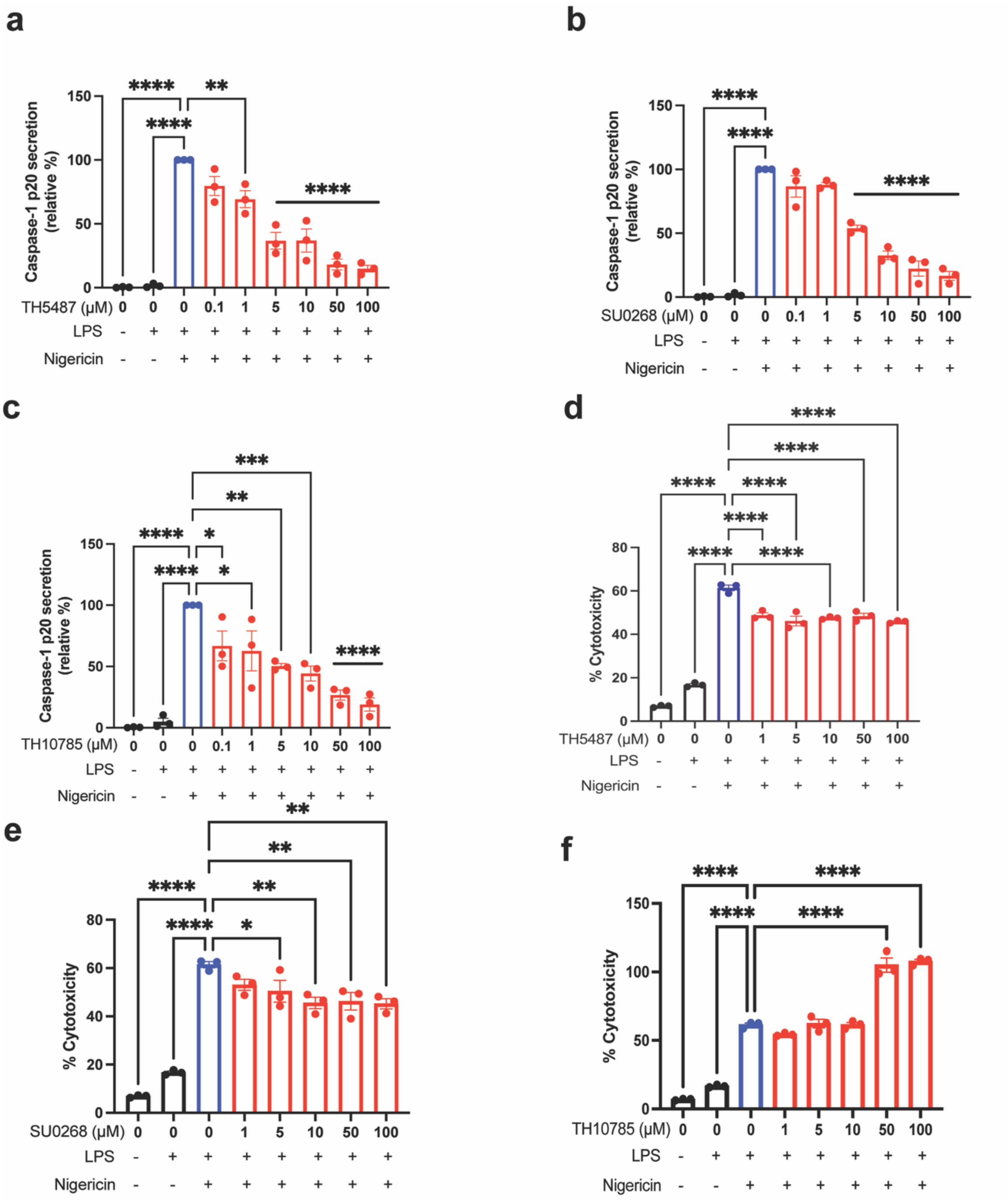

Figure. S3. Caspase-1 p20 section is and NLRP3-dependent cytotoxicity is inhibited by repurposed drugs in primary human. A-C) Primary human PBMCs were activated with LPS and nigericin and treated with TH5487, SU0268, or TH10785 at 0.1-100 µM. Casp1-p20 release was visualized by western (Fig.1 & 6) and quantified by relative band intensity (n = 3 biological replicates). (D-F) Primary human PBMCs were activated with LPS and nigericin and treated with small molecules from 1-100 µM. The amount of cytotoxicity was assessed by CyTox 96 fluorescence. A) TH5487 reduced inflammasome-related cytotoxicity from 10-100 µM. B) SU0268 reduced inflammasome-related cytotoxicity from 5-100 µM. C) TH10785 increased cytotoxicity at 50 and 100 µM. For all graphs, error bars signify the mean +/- SEM. The data was analyzed by one-way ANOVA with n = 3 biological replicates. p****<0.0001, p**<0.01, p*<0.05.

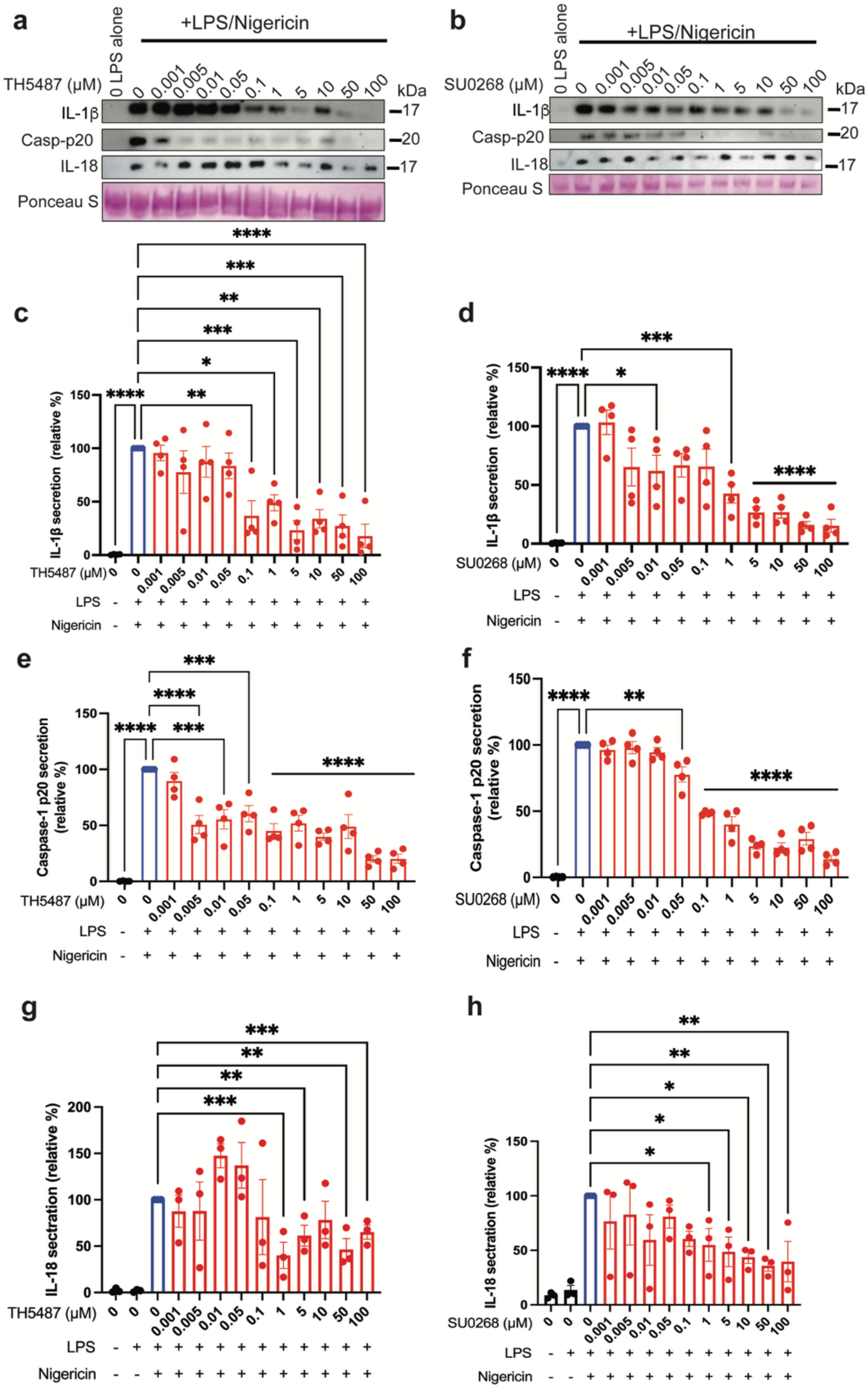

Figure. S4. IL-1β, Casp-p20, and IL-18 section is inhibited by TH5487 and SU0268 in immortalized human THP1s: Immortalized human THP1 cells were activated with LPS and nigericin and treated with TH5487 or SU0268 at 0.001-100 μM. A-B) IL-1β Casp-p20, and IL-18 release was visualized by western, and Ponceau stain was used as a loading control. C-D) IL-1β release was quantified by relative band intensity. (E-F) Casp-p20 release was quantified by relative band intensity. (G-H) IL-18 release was quantified by relative band intensity For all graphs, error bars signify the mean +/- SEM. The data was analyzed by one-way ANOVA with n = 3-4 biological replicates. p****<0.0001, p***<0.001, p**<0.01, p*<0.05.

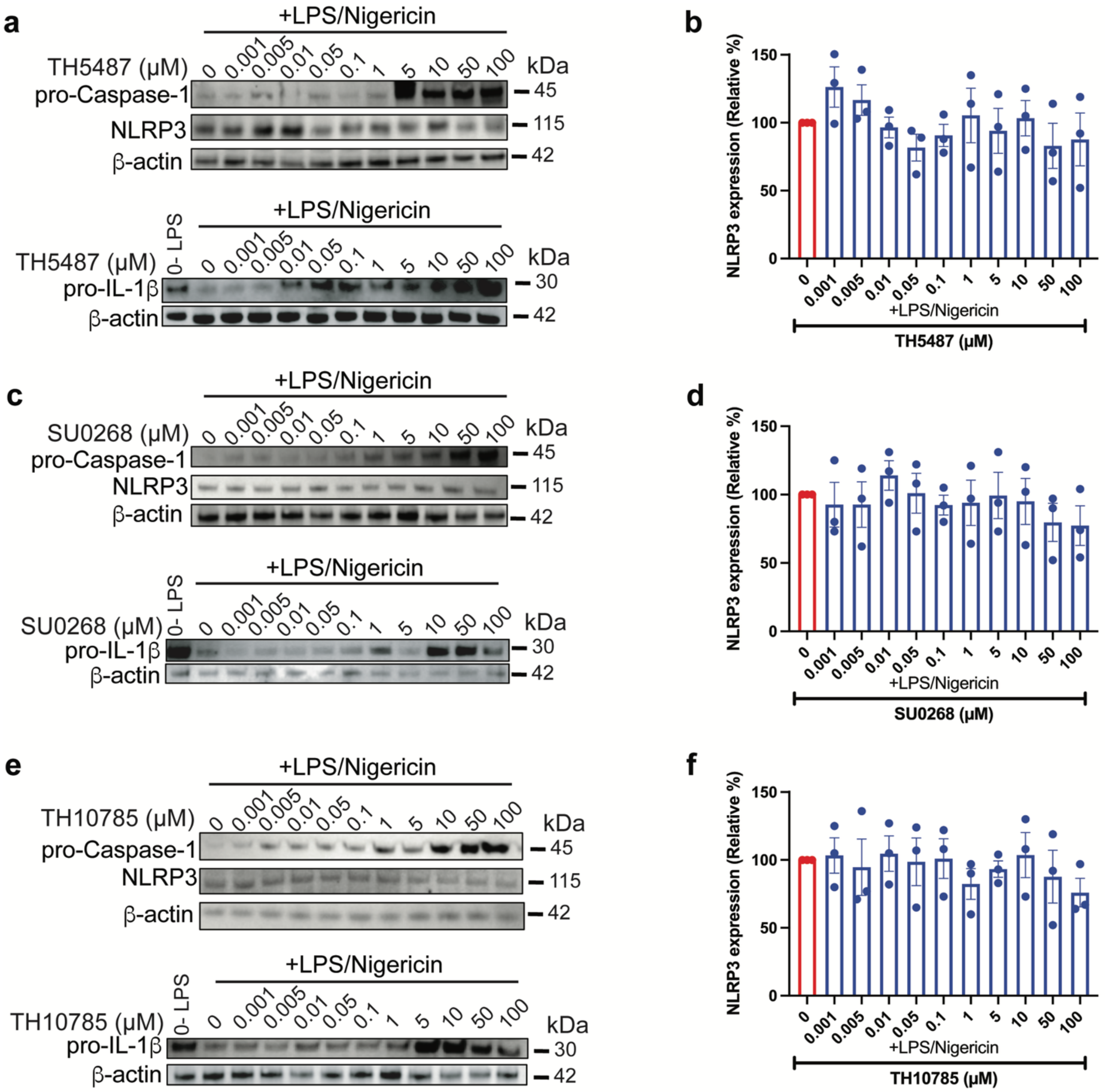

Figure. S5. Inhibitors increased the amount of Pro-IL-1β and Pro-Caspase-1 retained in the cell in human THP1 cells, matching the decrease in secretion of mature IL-1β and Caspase-1. A) Immortalized human THP1 cells were activated with LPS and nigericin and treated with TH5487 at 0.001-100 µM. Pro-IL-1β, Pro-Caspase-1, NLRP3, and b-actin expression were evaluated by western blot. B) NLRP3 expression under TH5487 treatment was quantified. C) Immortalized human THP1 cells were activated with LPS and nigericin and treated with SU0268 at 0.001-100 µM. Pro-IL-1β, Pro-Caspase-1, NLRP3, and b-actin expression were evaluated by western blot. D) NLRP3 expression under SU0268 treatment was quantified. E) Immortalized human THP1 cells were activated with LPS and nigericin and treated with TH10785 at 0.001-100 µM. Pro-IL-1β, Pro-Caspase-1, NLRP3, and b-actin expression were evaluated by western blot. F) NLRP3 expression under TH10785 was quantified. For all NLRP3 expression quantification, error bars signify the mean +/- SEM. The data was analyzed by one-way ANOVA with n = 3 biological replicates.

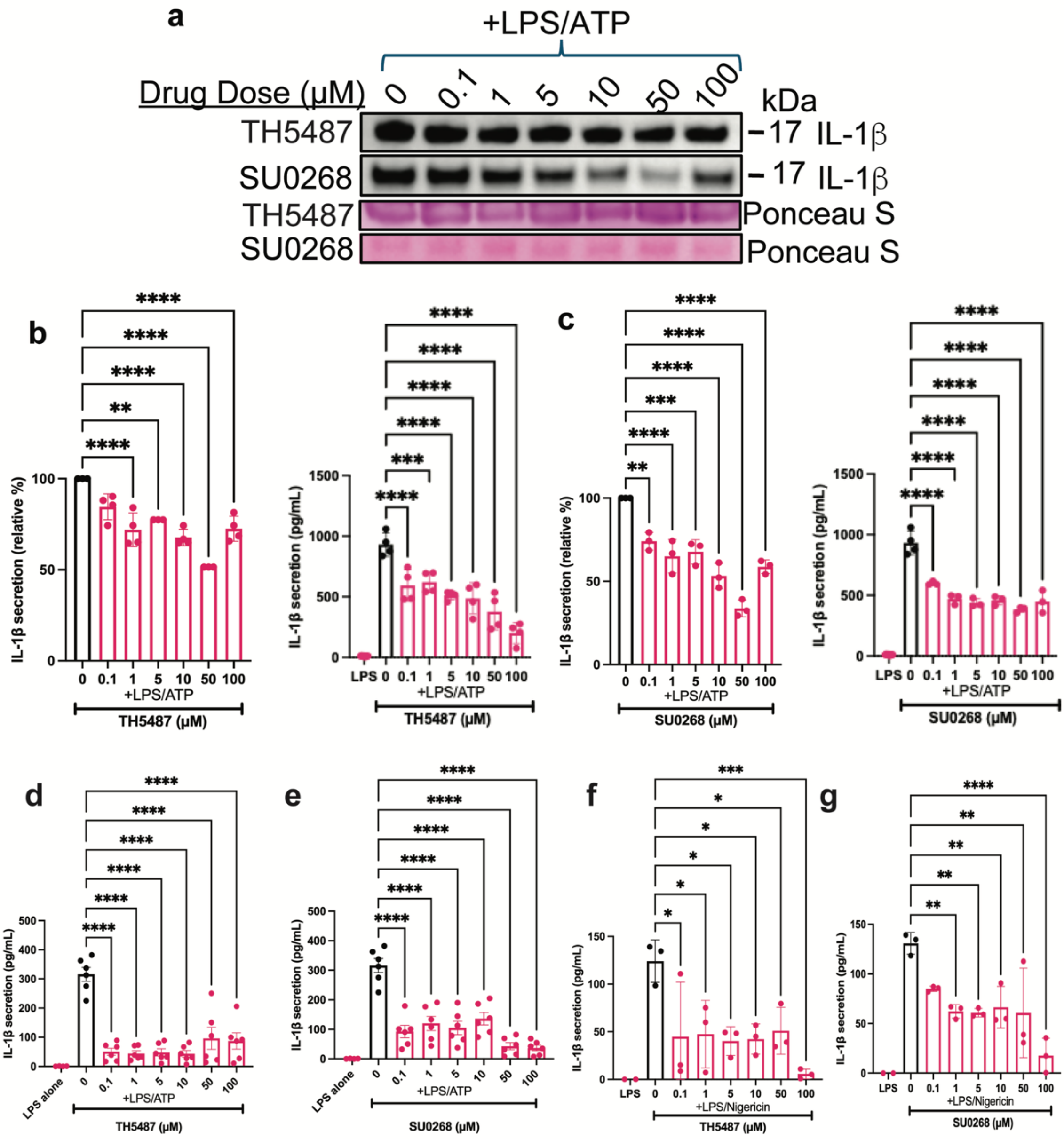

Figure. S6. TH5487 and SU0268 inhibit inflammasome activity in human THP1s and mouse iBMDMs activated with either LPS and ATP or LPS and nigericin. A) THP1 cells activated with LPS and ATP were treated with either TH5487 or SU0268 at 0.1-100 µM. Relative IL-1β release was visualized by western, and Ponceau stain was used as a loading control. B-C) Quantification of (A) and confirmation by ELISA for TH5487 data (n = 4 biological replicates). D) Mouse iBMDMs were primed and activated with LPS and ATP and treated with 0.1-100 µM TH5487. The amount of IL-1β secreted into the media was measured using an IL-1β ELISA (n = 6 biological replicates). E) Mouse iBMDMs were primed and activated with LPS and ATP and treated with 0.1-100 µM SU0268. The amount of IL-1β secreted into the media was measured using an IL-1β ELISA (n = 6 biological replicates). F) Mouse iBMDMs were primed and activated with LPS and nigericin and treated with 0.1-100 µM TH5487. The amount of IL-1β secreted into the media was measured using an IL-1β ELISA (n = 3 biological replicates). G) Mouse iBMDMs were primed and activated with LPS and nigericin and treated with 0.1-100 µM SU0268. The amount of IL-1β secreted into the media was measured using an IL-1β ELISA (n = 3 biological replicates). For all graphs, error bars signify the mean +/- SEM. The data was analyzed by one-way ANOVA with p****<0.0001, p**<0.01, p*<0.05.

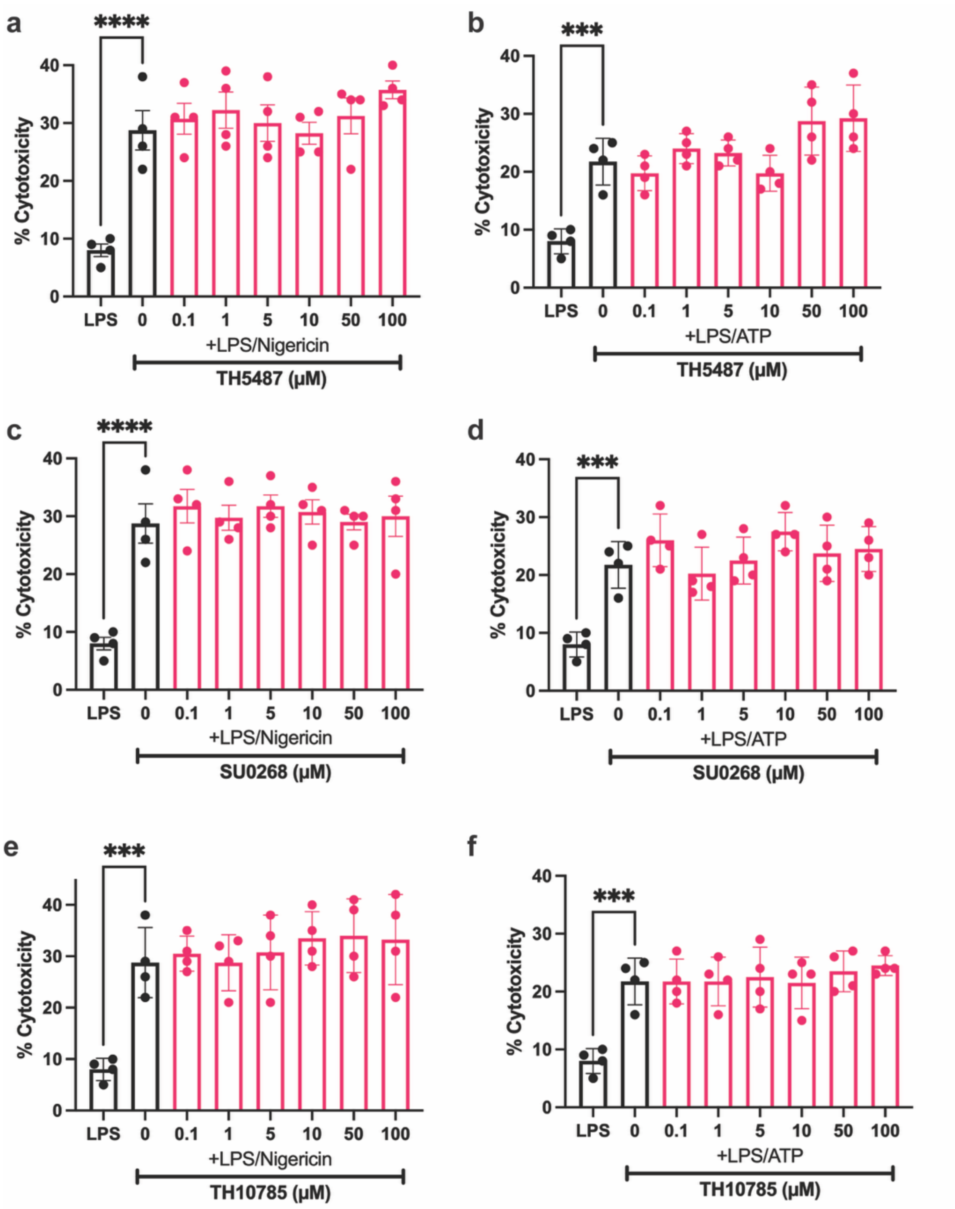

Figure. S7. Low cytotoxicity with hOGG1 small molecules in LPS/ATP- or LPS/nigericin-stimulated THP-1 cells. A) Human monocyte THP1 cells were activated with LPS and nigericin and treated with TH5487 from 0.1-100 µM. The cell death was quantified by the presence of lactose dehydrogenase (LDH) in the supernatant of treated cells. B) Human monocyte THP1 cells were activated with LPS and ATP and treated with TH5487 from 0.1-100 µM. The cell death was quantified by the presence of lactose dehydrogenase (LDH) in the supernatant of treated cells. C) Human monocyte THP1 cells were activated with LPS and nigericin and treated with SU0268 from 0.1-100 µM. The cell death was quantified by the presence of lactose dehydrogenase (LDH) in the supernatant of treated cells. D) Human monocyte THP1 cells were activated with LPS and ATP and treated with SU0268 from 0.1-100 µM. The cell death was quantified by the presence of lactose dehydrogenase (LDH) in the supernatant of treated cells. E) Human monocyte THP1 cells were activated with LPS and nigericin and treated with TH10785 from 0.1-100 µM. The cell death was quantified by the presence of lactose dehydrogenase (LDH) in the supernatant of treated cells. F) Human monocyte THP1 cells were activated with LPS and ATP and treated with TH10785 from 0.1-100 µM. The cell death was quantified by the presence of lactose dehydrogenase (LDH) in the supernatant of treated cells. For all graphs, error bars signify the mean +/- SEM. The data was analyzed by one-way ANOVA with n = 4 biological replicates. p****<0.0001, p***<0.001

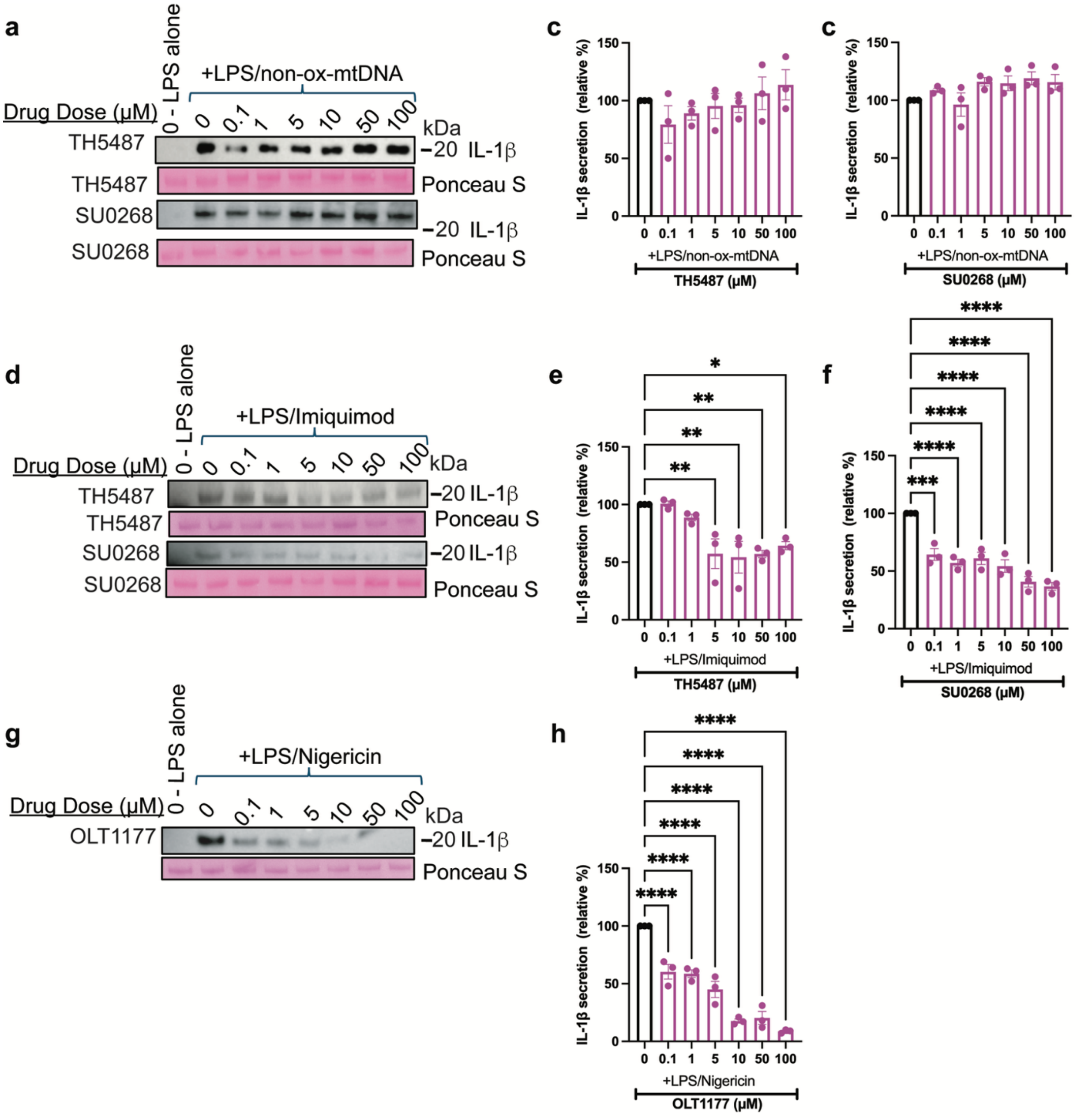

Figure S8: TH5487 and SU0268 are specific inhibitors of NLRP3 under a variety of activating conditions. A) The AIM2 inflammasome was activated in human monocyte THP1 cells with LPS and transfected non-ox-mtDNA. Between priming and activation, cells were treated with from 0.1-100 μM of TH5487 and SU0268. Relative IL-1β release was visualized by western, and Ponceau stain was used as a loading control. C) Quantification of TH5487 data from (A). C) Quantification of SU0268 data from (A). D) NLRP3 was activated in human monocyte THP1 cells with LPS and Imiquimod. Between priming and activation, cells were treated with from 0.1-100 μM of TH5487 and SU0268. Relative IL-1β release was visualized by western, and Ponceau stain was used as a loading control. E) Quantification of TH5487 data from (B). F) Quantification of SU0268 data from (B). G) NLRP3 was activated in human monocyte THP1 cells with LPS and Nigericin. Between priming and activation, cells were treated with from 0.1-100 μM of OLT1177. Relative IL-1β release was visualized by western, and Ponceau stain was used as a loading control. H) Quantification of data from (G).

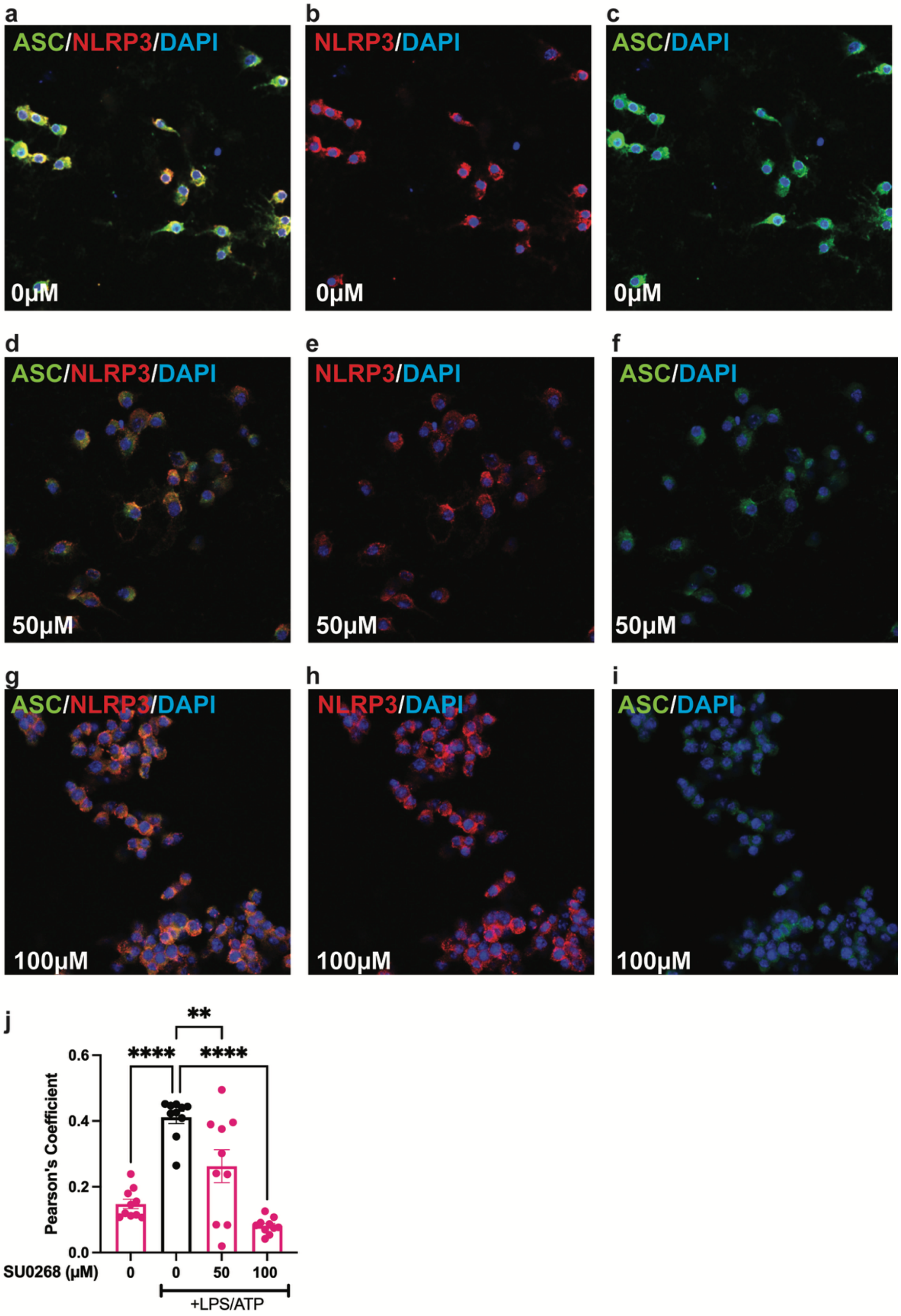

Figure. S9: Full-scale images from immunofluorescence data in Fig. 2. A-C) iBMDMs were treated with LPS and ATP and 0 μM SU0268 then probed for ASC (A), NLRP3 (B), or both (C). D-F) iBMDMs were treated with LPS and ATP and 50 μM SU0268 then probed for ASC (D), NLRP3 (E), or both (F). G-I) iBMDMs were treated with LPS and ATP and 100 μM SU0268 then probed for ASC (G), NLRP3 (H), or both (I). J) The Pearson's coefficient of the colocalization of ASC and NLRP3 signal when the cells were primed with LPS only, challenged with 0, 50, or 100 μM SU0268 and activated with ATP, and >1000 cells were counted per condition prior to statistical analysis. Error bars signify mean +/- SEM, p** = 0.0044, p****<0.0001.

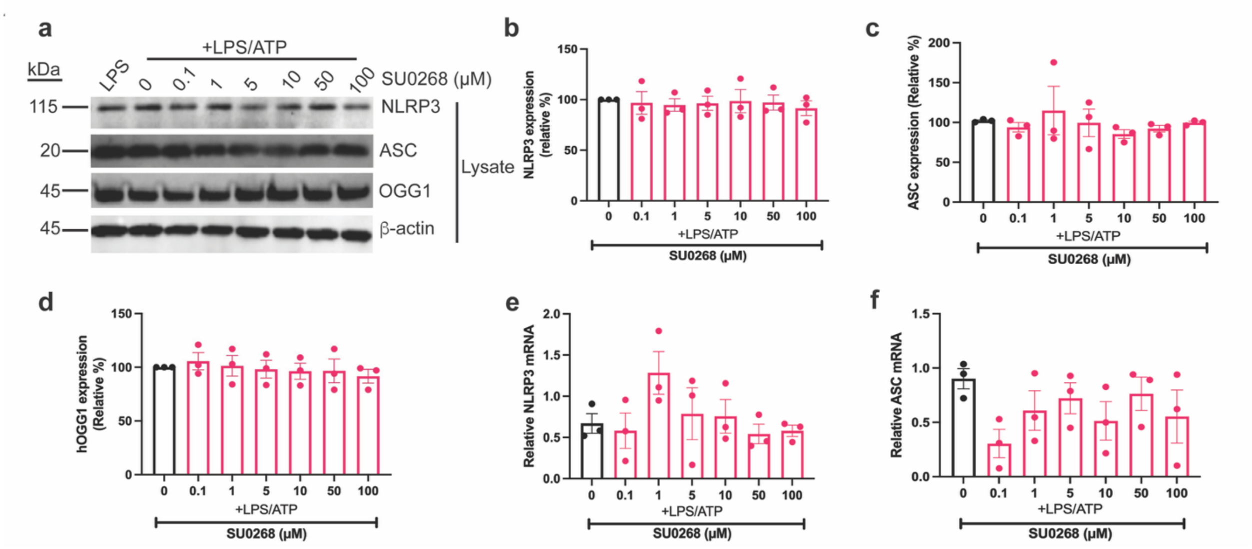

Figure. S10. ASC speck and inflammasome assembly inhibition do not effect protein expression. A) iBMDMs were primed with LPS and activated with ATP and treated with SU0268. The lysate of the cells were probed with antibodies for NLRP3, ASC, OGG1, and β-actin and visualized by western blot. The band intensities of western blots against NLRP3 (B), ASC (C), and OGG1 (D) were quantified and compared to the untreated activated control. RNA was isolated from treated cells and RT-PCR followed by qPCR was performed to quantify mRNA expression for either NLRP3 (E) or ASC (F). For all graphs, error bars signify the mean +/- SEM. The data was analyzed by one-way ANOVA with n = 3 biological replicates.

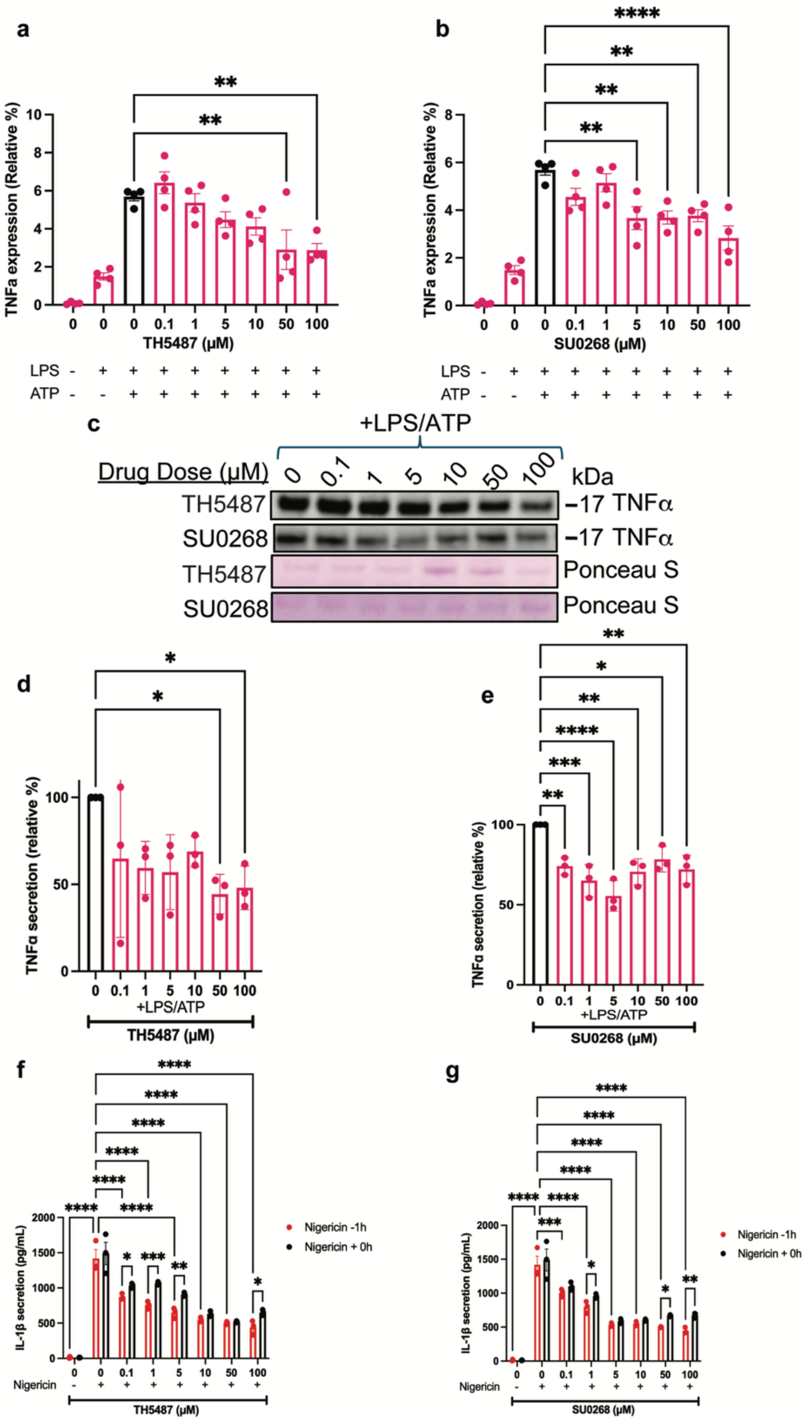

Figure. S11. TH5487 and SU0268 have some effect on inflammatory priming activity in human THP1 cells. THP1 cells activated with LPS and ATP were treated with either TH5487 or SU0268 at 0.1-100 µM. A) The expression of TNF-α in cells treated with TH5487 was evaluated by qPCR (n = 4 biological replicates). B) The expression of TNF-α in cells treated with SU0268 was evaluated by qPCR (n = 4 biological replicates). C) Relative TNF-α release was visualized by western, and Ponceau stain was used as a loading control. D) Quantification of (C) for TH5487 data (n = 3 biological replicates). E) Quantification of (C) for SU0268 data (n = 3 biological replicates). F) THP1 cells activated primed with LPS then treated with the TH5487 or SU0268 from 0.1-100 µM 1 hour before or during the1 hour activation by Nigericin. IL-1b secretion was evaluated by ELISA (n = 3 biological replicates). For all graphs, error bars signify the mean +/- SEM. The data was analyzed by one-way ANOVA with p****<0.0001, p***<0.001, p**<0.01, p*<0.05.

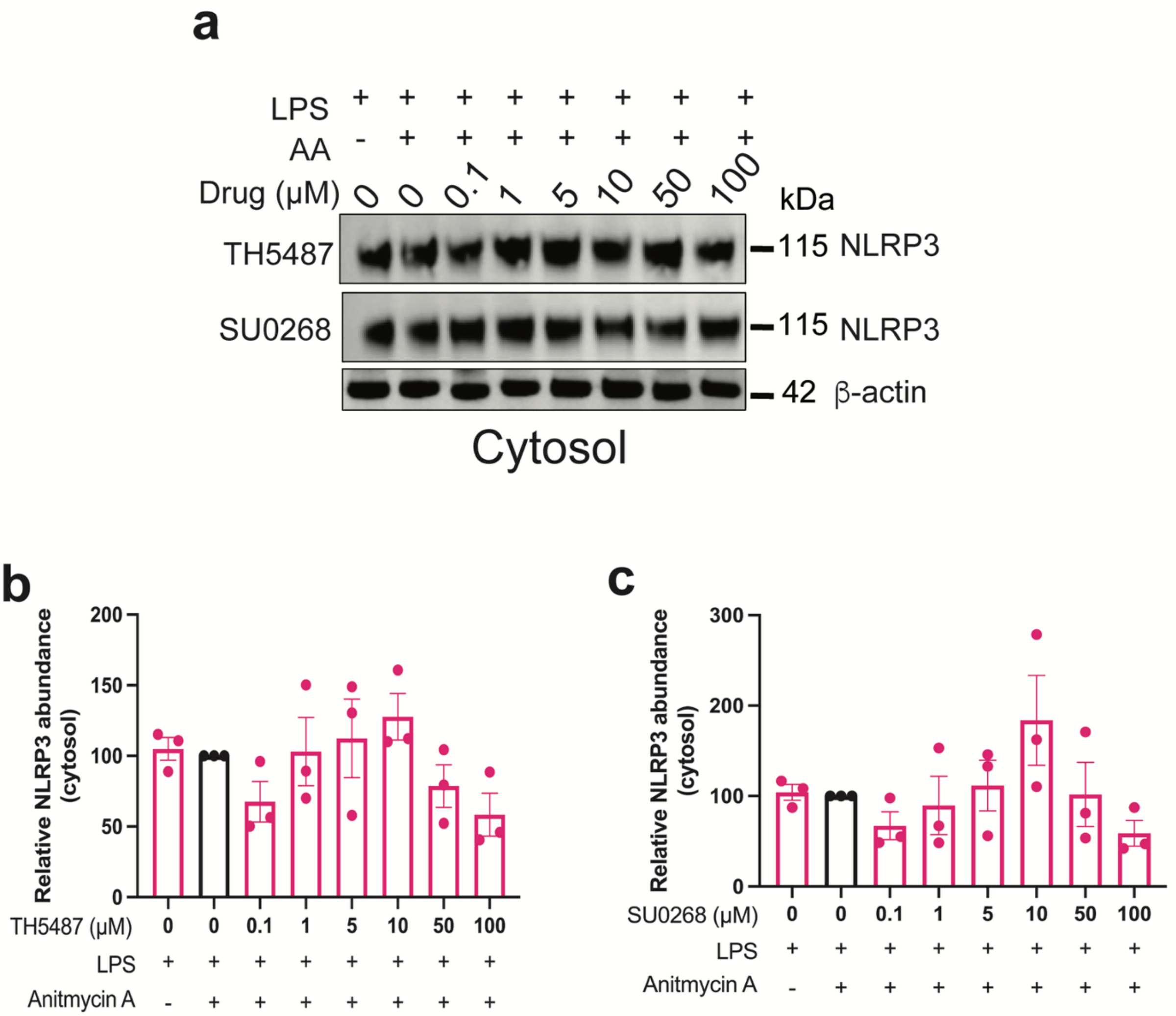

Figure. S12. During mitochondrial dysfunction and subsequent treatment with small molecules, the majority of NLRP3 remains in the cytosol. A) THP1 cells activated with LPS and Antimycin A were treated with either TH5487 or SU0268 at 0.1-100 µM. The cytosolic fraction was isolated and the relative amount of NLRP3 in the cytosolic fraction was visualized by western blot. B) quantification of (A) for TH5487. C) Quantification of (A) for SU0268. For all graphs, error bars signify the mean +/- SEM. The data was analyzed by one-way ANOVA with n = 3 biological replicates.

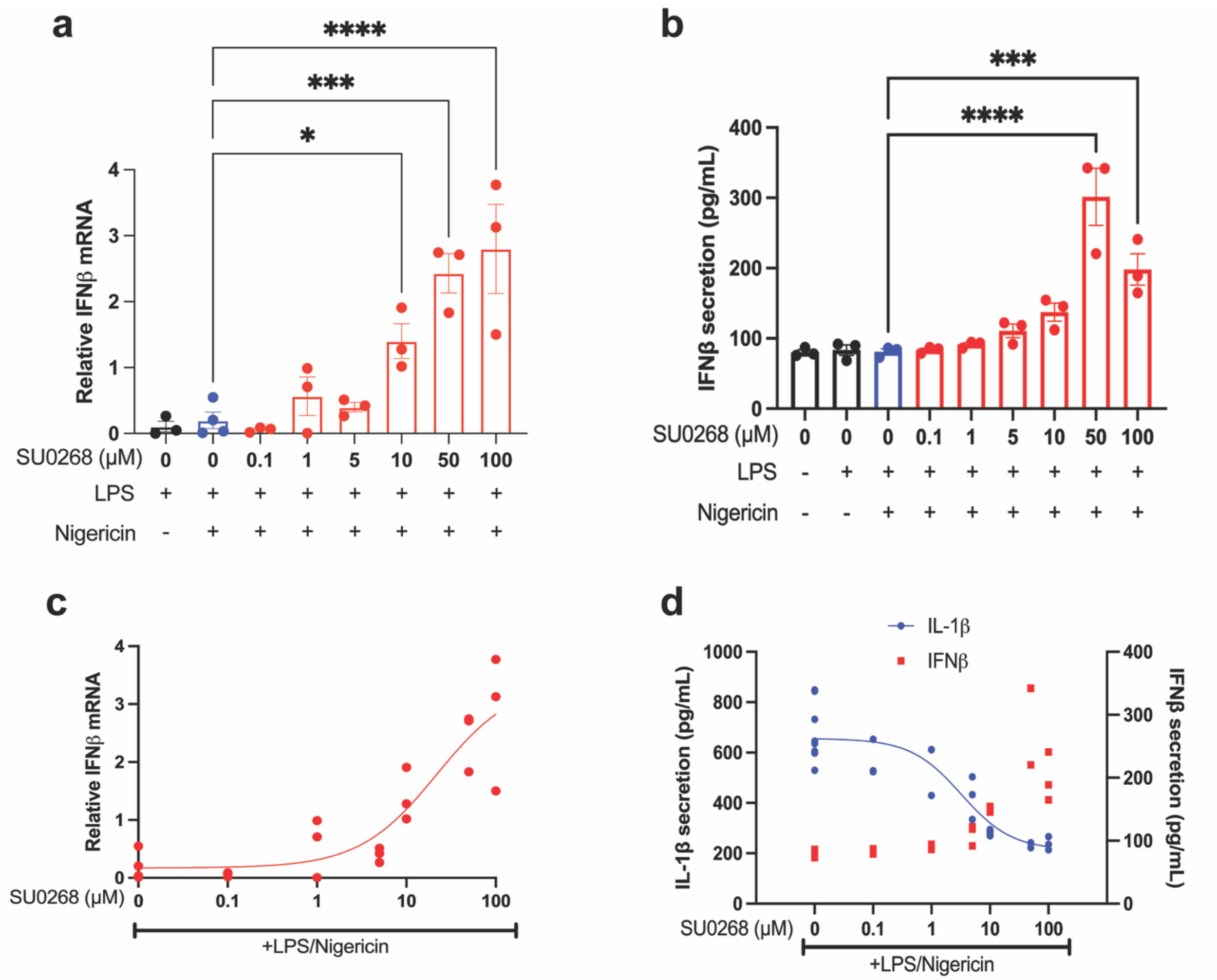

Figure. S13. SU0268 inhibits NLRP3 activity and activates the cGAS-STING pathway in primary human PBCMs. Primary human PBMCs from healthy donors were activated with LPS and nigericin and treated with SU0268 at 0.1-100 μM. A) IFN-β expression was quantified by qPCR (n = 3 biological replicates). B) IFN-β secretion was quantified by ELISA (n = 3 biological replicates). C) The EC50 of SU0268 for IFN-β was found using non-linear regression at 21.92 μM with R^2 = 0.81. D) IL-1β (blue) and IFN-β (red) secretion was quantified by ELISA and compared using non-linear regression showing inversely related trends of expression (n = 5 biological replicates). $IC50_{SU0268}$: 3.25 μM, R^2_{SU0268}: 0.8209. For bar graphs, error bars signify the mean +/- SEM. For the bar graphs, the data was analyzed by one-way ANOVA with p****<0.0001, p***<0.001, p*<0.05. For the line graph, error bars signify mean +/- SD and the data was analyzed using non-linear regression.

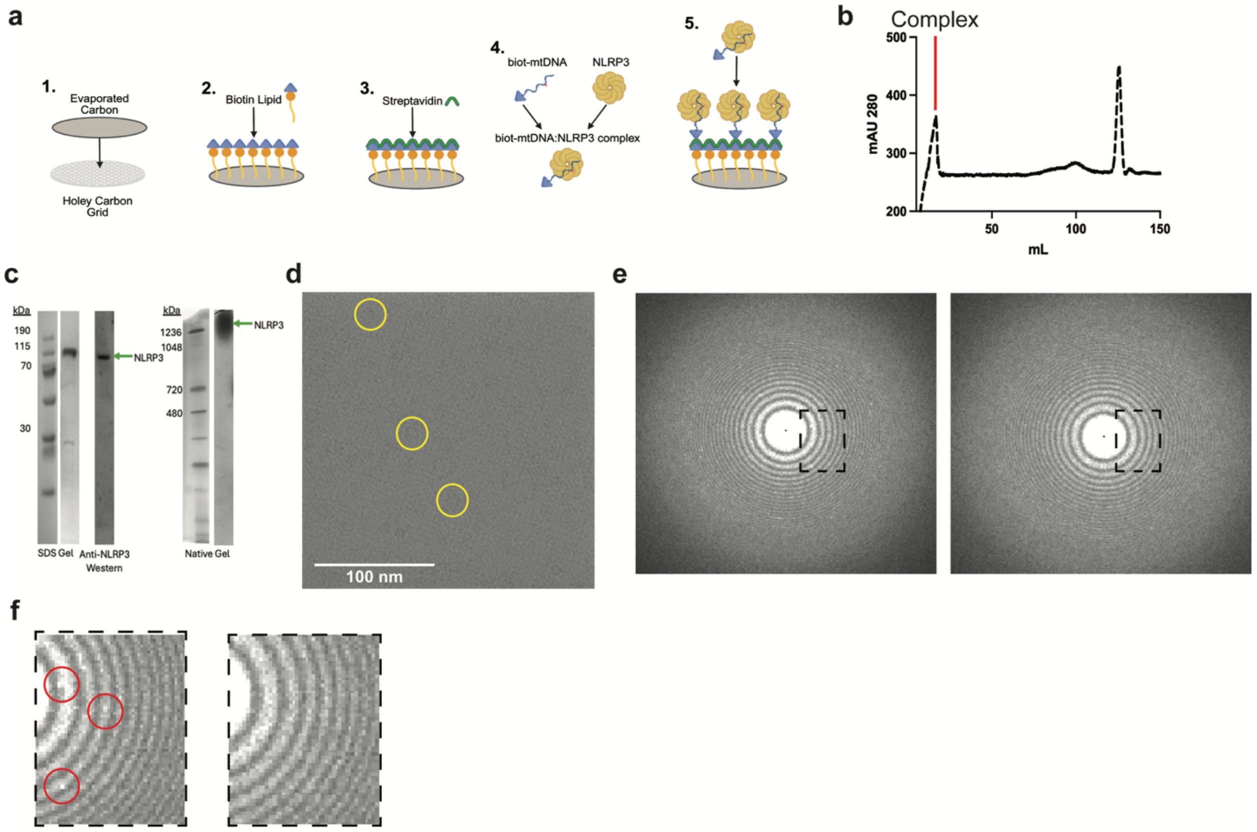

Figure. S14. NLRP3 purified in the presence of TH5487 can be visualized on streptavidin-affinity grids using cryo-EM. A) Schematic of streptavidin grid preparation workflow B) Full length human NLRP3 was expressed in Expi293 cells in the presence of TH5487 and purified by affinity and size exclusion chromatography. The SEC trace of from the purification of the NLRP3:TH5487 complex is shown. C) SDS Page gel and western blot from the NLRP3:TH5487 purification. A band associated with NLRP3 is shown at the expected size of ~120 kDa. On a native gel, a band associated with an NLRP3 decamer is shown at the expected size of ~1200 kDa. D) Example cryoEM micrograph before streptavidin lattice removal. Yellow circles show NLRP3 particles bound to the streptavidin/biot-DNA layer. E) FFT of un-subtracted (left) or streptavidin lattice subtracted (right) micrographs. F) Zoomed in view of dashed boxes in (E). Left: un-subtracted micrograph, right: streptavidin lattice subtracted micrograph. Red circles indicate speckle contamination.

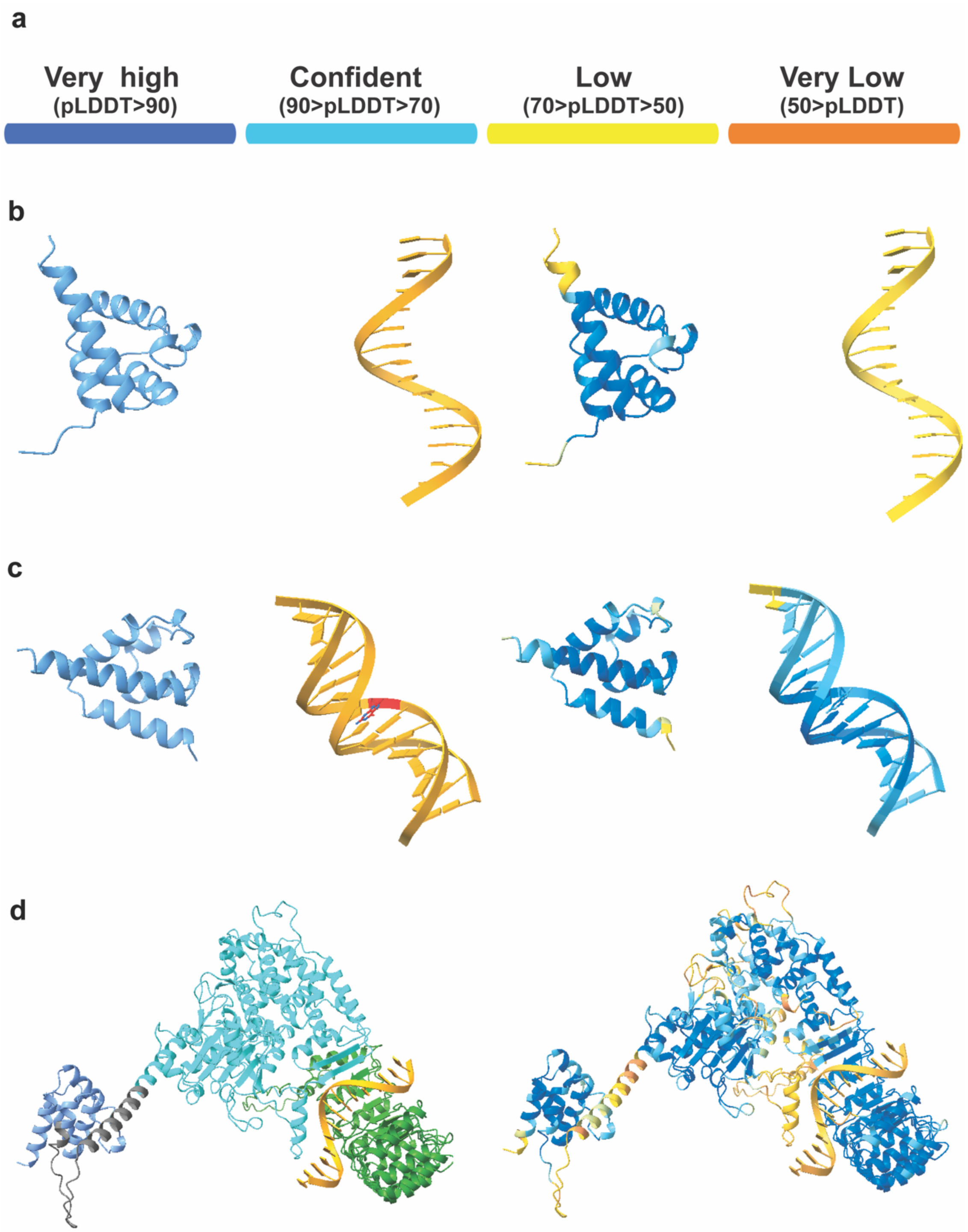

Figure. S15. AlphaFold3 does not predict the NLRP3 pyrin domain will interact with single stranded non-oxidized DNA or double stranded oxidized DNA, and that FL NLRP3 might bind non-oxDNA in the NACHT/LRR A) Key for AlphaFold confidence color code. B) Left: AlphaFold model of the NLRP3 pyrin domain (residues 1-85, blue) in the presence of single stranded non oxidized DNA (orange). Right: the same model colored by confidence score. PTM = 0.7, iPTM = 0.05 C) Left: AlphaFold model of the NLRP3 pyrin domain (residues 1-85, blue) in the presence of double stranded (orange) oxidized DNA (red, from PDBID: 1EBM). Right: the same model colored by confidence score. PTM = 0.85, iPTM = 0.24. D) Left: AlphaFold model of the full-length NLRP3 (pyrin: blue, NACHT: cyan, LRR: green) in the presence of single-stranded non-oxidized DNA (orange). Right: the same model colored by confidence score. PTM = 0.76, iPTM = 0.75.

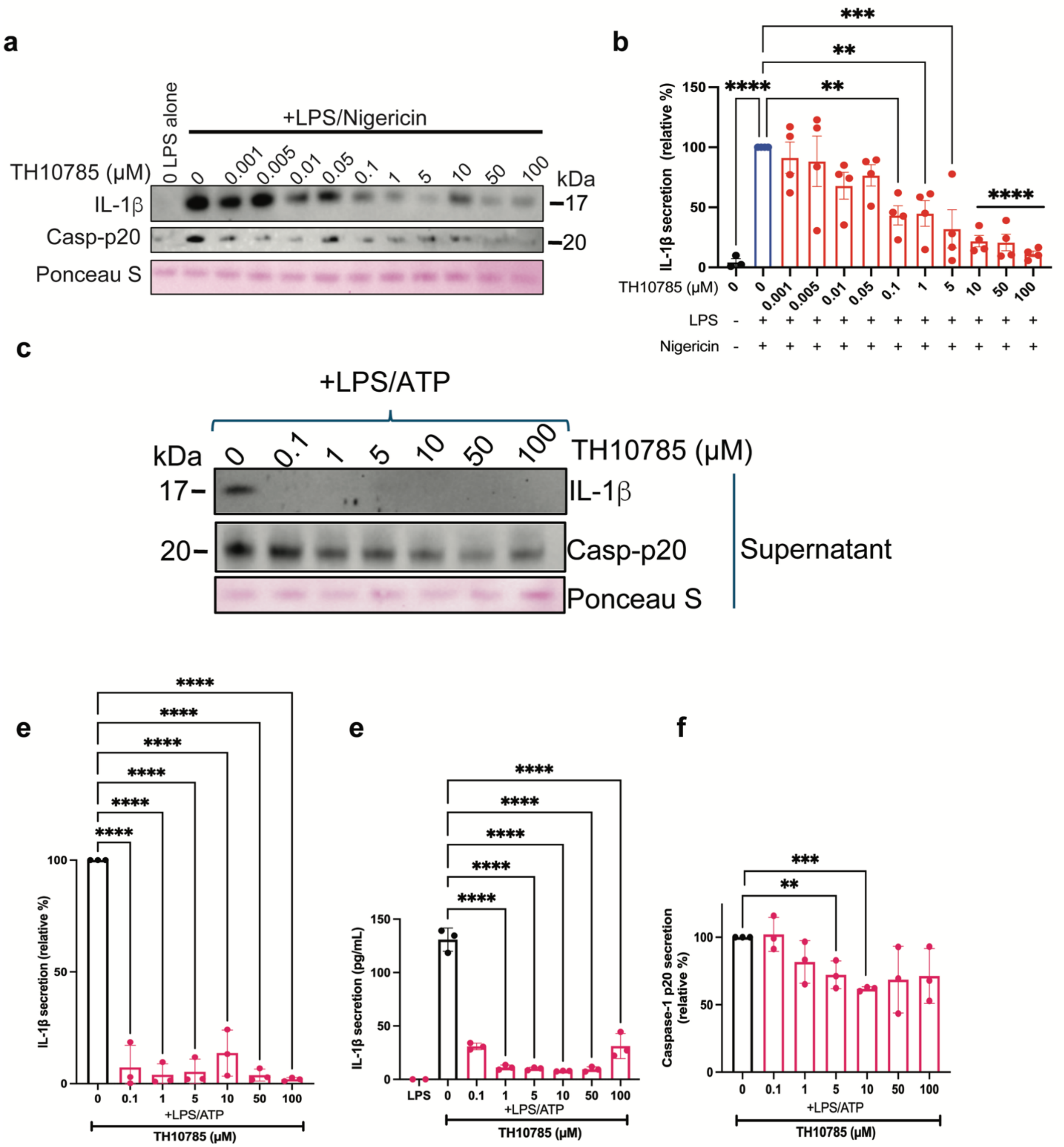

Figure. S16. TH10785 inhibits inflammasome activation in mouse macrophages. Immortalized human THP1 cells were activated with LPS and nigericin and treated with TH10785 at 0.001-100 μM. (A) IL-1β release was visualized by western, and Ponceau stain was used as a loading control. (B) IL-1β release was quantified by relative band intensity (n = 4 biological replicates). (C) Mouse iBMDMs activated with LPS and ATP were treated with TH10785 at 0.1-100 μM. The amounts of secreted IL-1β and Casp-p20 were visualized by western blot. (D/E) The amount of secreted IL-1β was quantified both by western blot (D) and ELISA (E) (n = 3 biological replicates). (F) The amount of secreted Casp-p20 was quantified by western blot (n = 3 biological replicates). For all graphs, error bars signify the mean +/- SEM. The data was analyzed by one-way ANOVA with p****<0.0001, p***<0.001, p**<0.01.

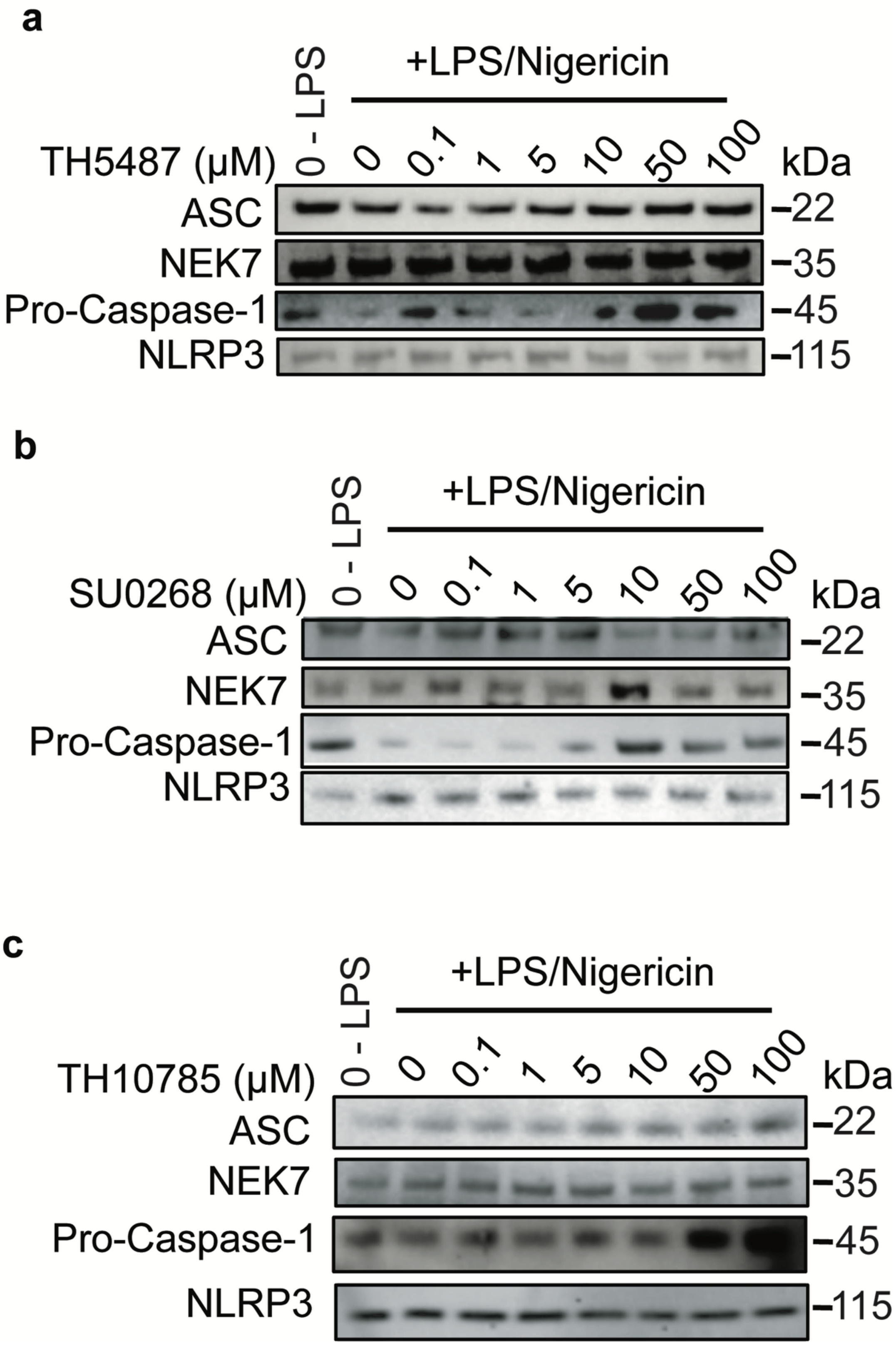

Figure. S17. Whole cell lysate input samples that were used for co-IP analysis.

**Cell type**	CD45+	CD14+	CD19+	CD56+	CD3+	CD3+CD4+	CD3+CD8+	Viability
**% of population**	99.20	40.80	8.20	36.20	27.40	47.70	42.80	97
**Phenotype**	Leukocyte	Monocyte	B-cell	NK-cell	T-cell	Th-cell	Tc-cell	Living

Table S1. Morphology of primary human PBMC cell types.

WT NLRP3: AA codes	hOGG1: AA codes	Distance (Å) hOGG1 TH10785/WT	Distance (Å) hOGG1 TH10785/Model
Lys 2	Glu/Lys 249		0.632
Ala 4	Ala 251	10.556	0.439
Cys 8	Cys 255	7.906	0.373
Ala 11	Ala 258	16.110	0.286
Asp 16	Asp 268	3.571	17.863
Asp 19	Asp 268	7.782	13.315
Val 20	Val 267	13.134	6.447
Asp 21	Asp 268	8.048	7.031
His 28	His 270	10.53	5.463
Asp 31	Asp 278	3.378	4.847
Tyr 32	Tyr 279	6.641	4.436
Pro 34	Pro 283	24.239	2.473
Pro 42	Pro 291	17.71	11.260
Gln 45	Gln 294	13.188	0.634
Thr 46	Thr 295	9.240	0.637
Leu 54	Leu 299	0.420	0.378
Gly 63	Gly 308	0.528	0.101
Trp 68	Trp 313	3.621	0.098
Ala 69	Ala 314	1.392	0.176
Ala 71	Ala 316	4.595	0.571
Val 72	Val 317	3.926	0.793
Phe 75	Phe 319	1.991	0.649
Ala 77	Ala 321	3.995	0.949
Arg 80	Arg 324	9.094	1.279

Table S2. Measured distanced between the NLRP3 pyrin structure or the NLRP3 pyrin domain model and hOGG1 bound to TH10785 presented in Fig.5. The locations of active site amino acids in hOGG1 bound to TH10785 (PDB: 7AYY, 249-325) were compared to that of similar amino acids in the NLRP3 pyrin domain (PDB: 7PZC, 1-91) (third from right column) or the SWISS-MODEL generated model of the NLRP3 pyrin domain (far left column).

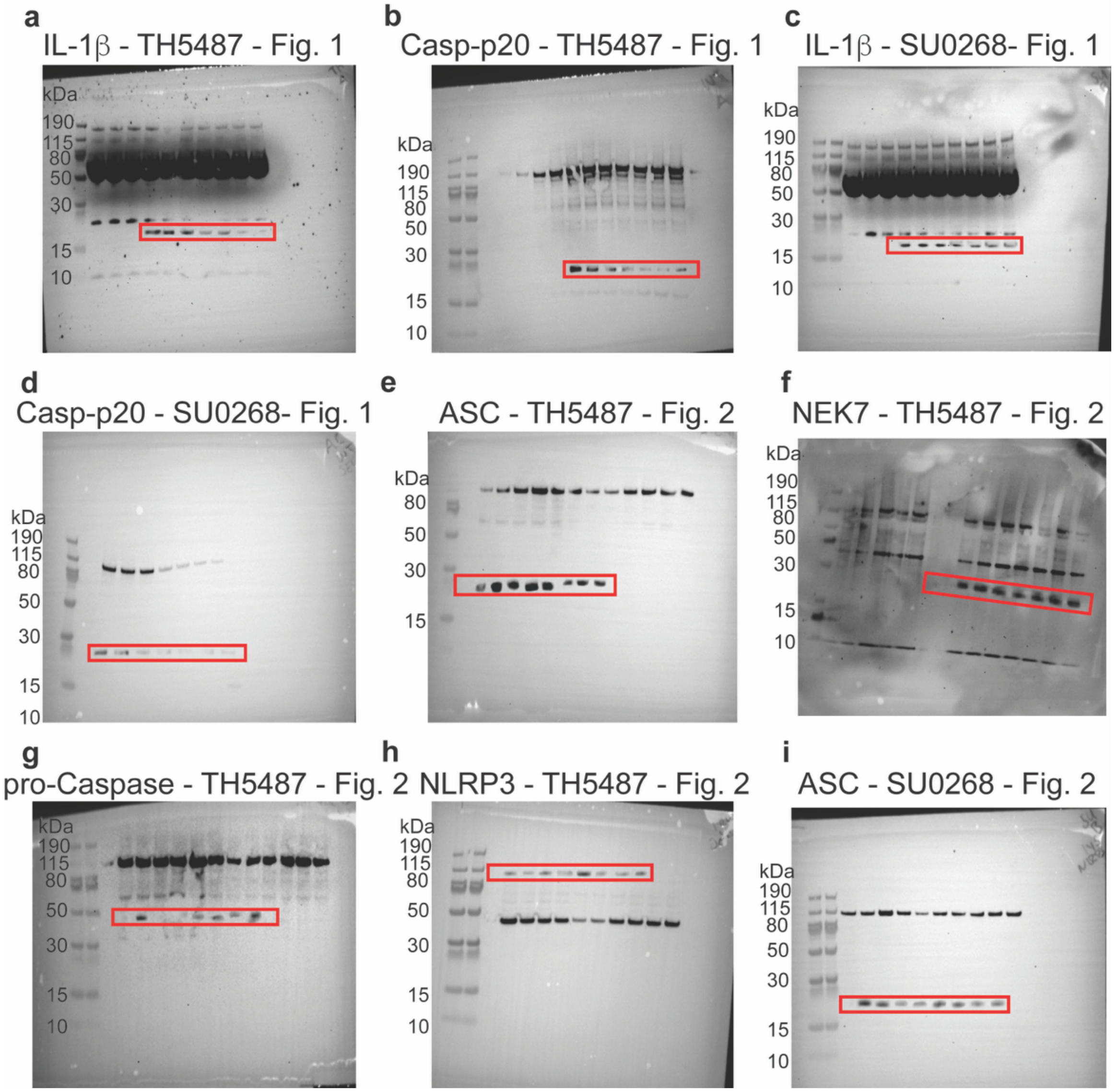

Figure S18: Uncropped blots from Figures 1 and 2. A-D) Figure 1 blots. E-I) Figure 2 blots

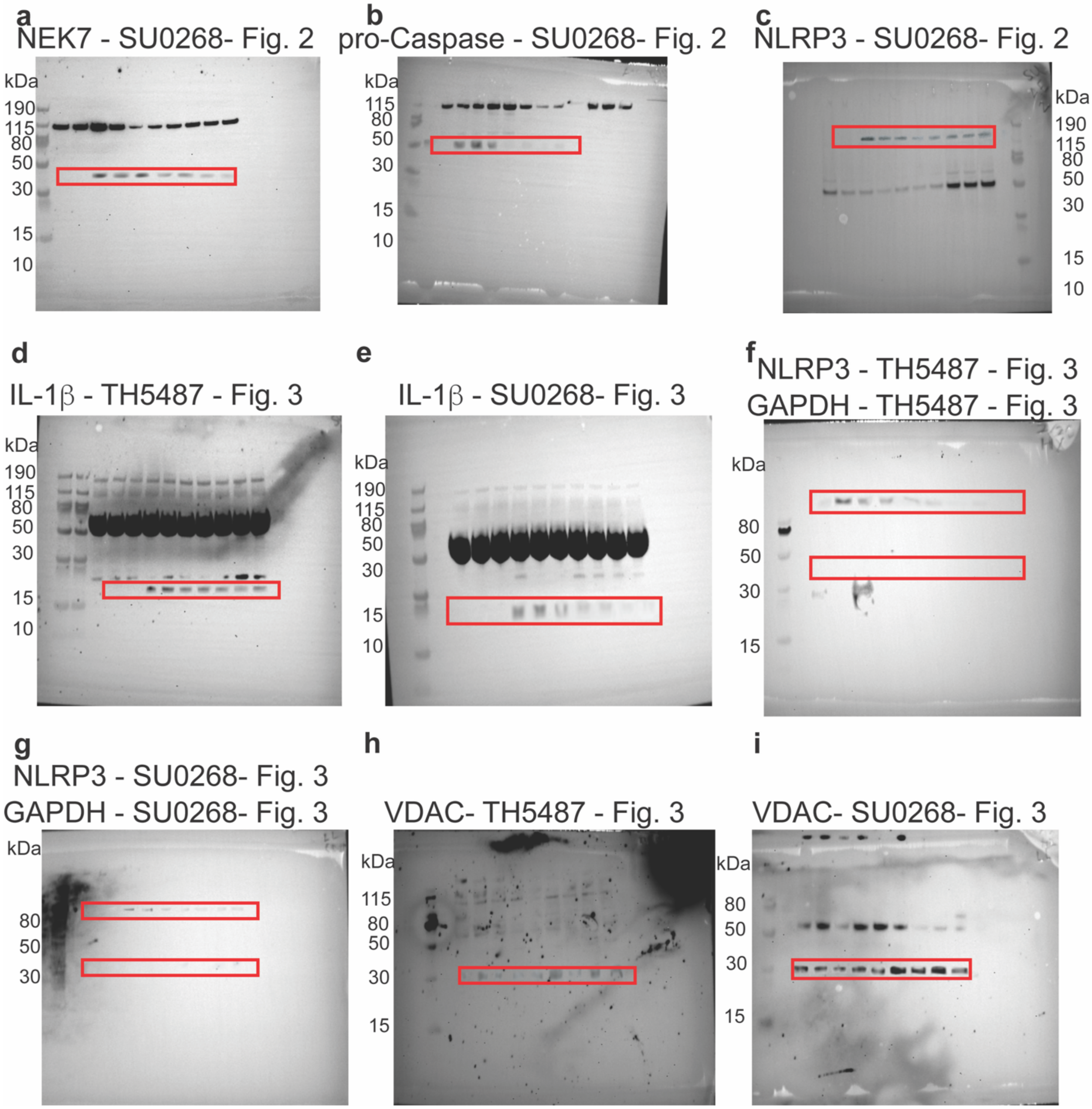

Figure S19: Uncropped blots from Figures 2 and 3. A-C) Figure 2 blots. D-I) Figure 3 blots

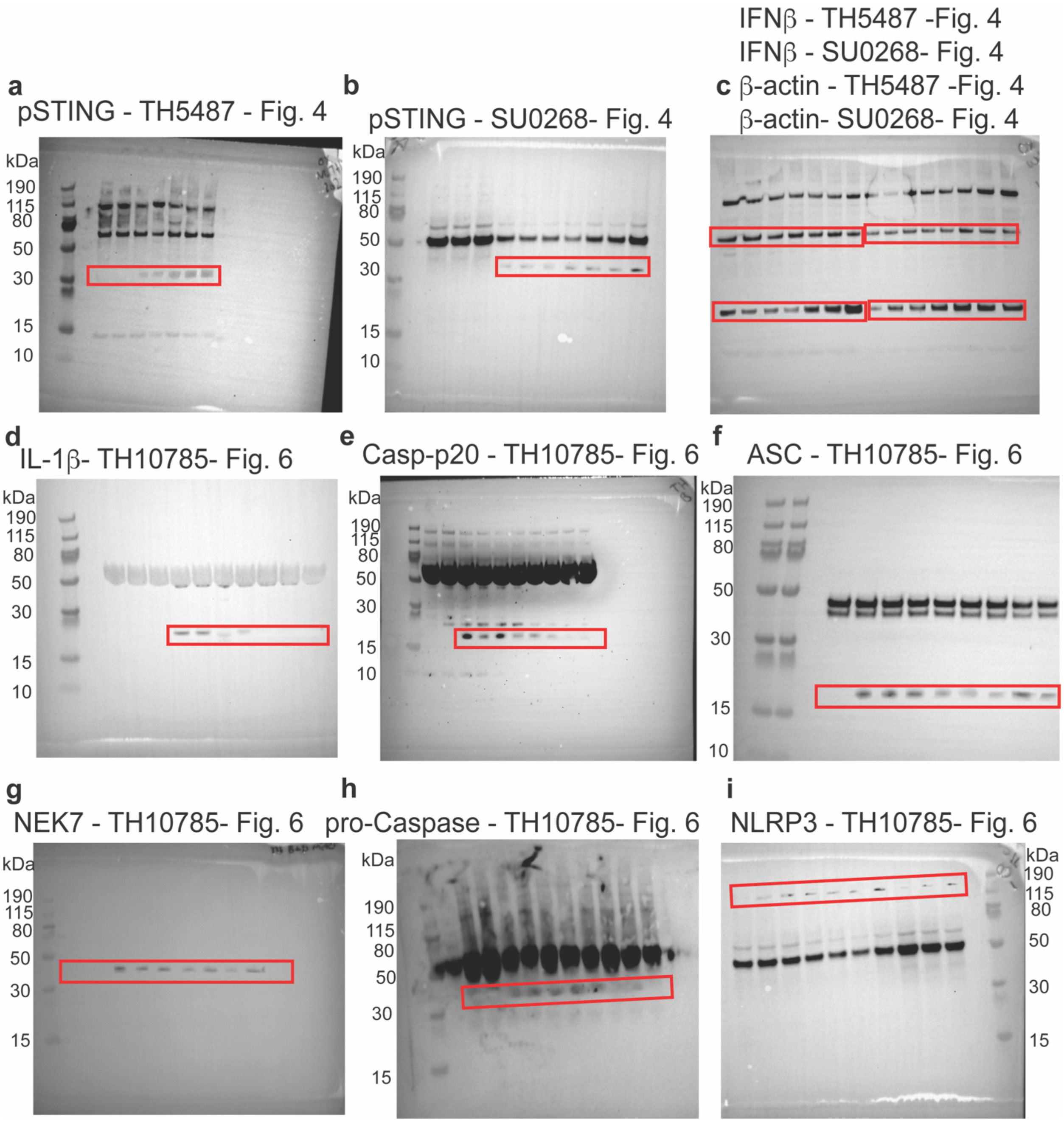

Figure S20: Uncropped blots from Figures 4 and 6. A-C) Figure 4 blots. D-I) Figure 6 blots

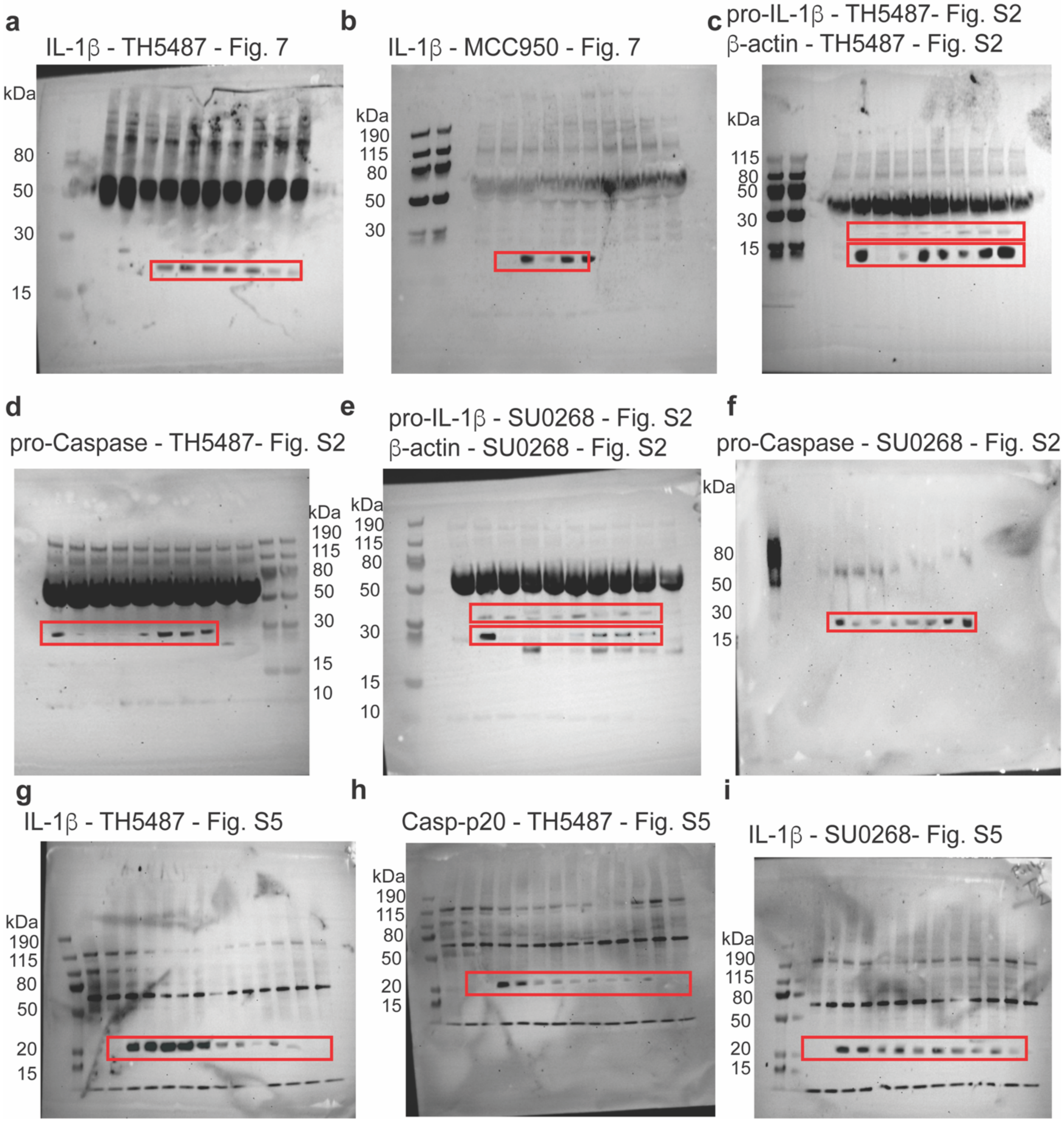

Figure S21: Uncropped blots from Figures 7, S2, and S5. A-B) Figure 7 blots. C-F) Figure S2 blots. G-I) Figure S5 blots

5

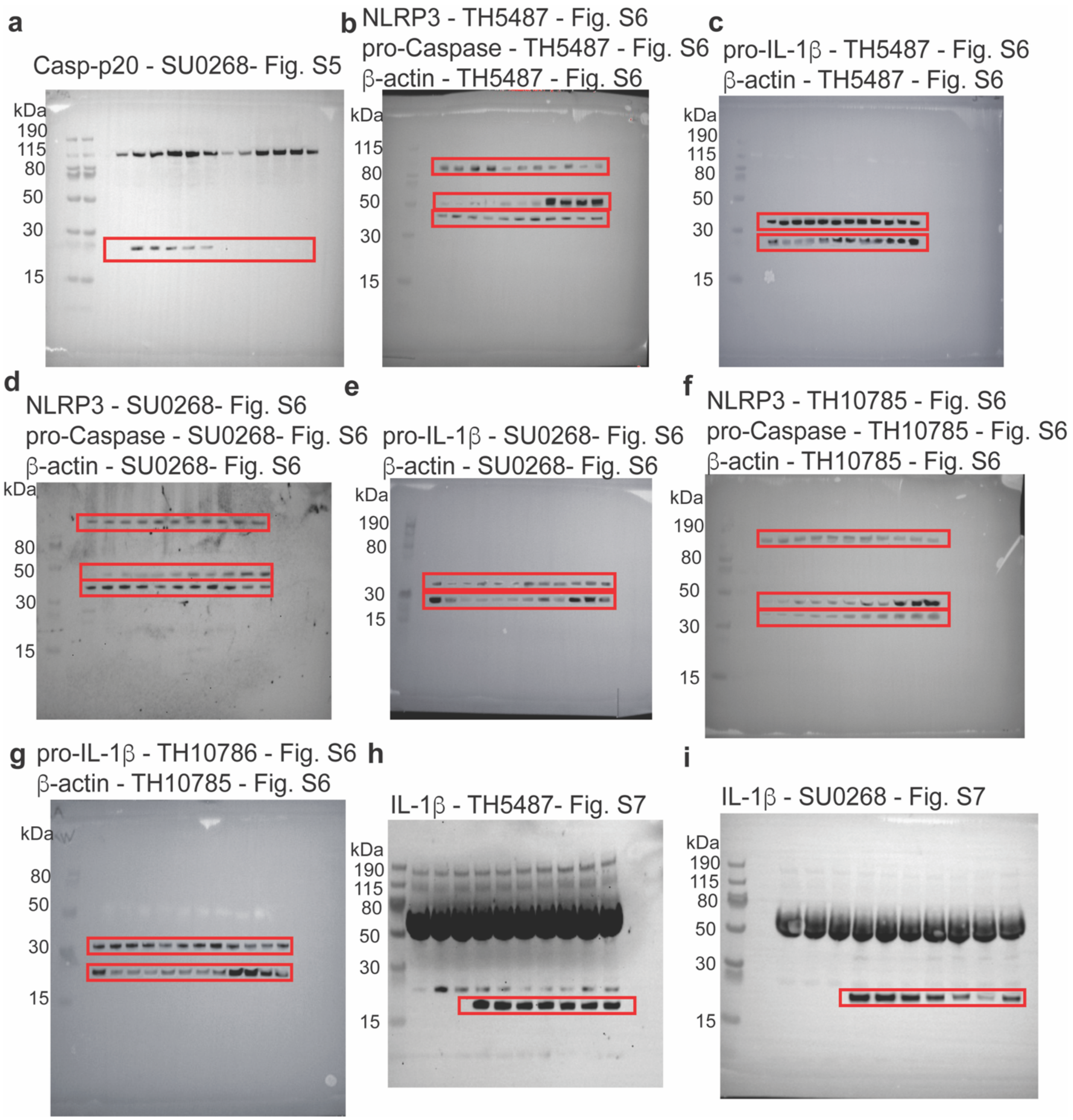

Figure S22: Uncropped blots from Figures S5, S6, and S7. A) Figure S5 blot. B-G) Figure S6 blots. H-I) Figure S7 blots

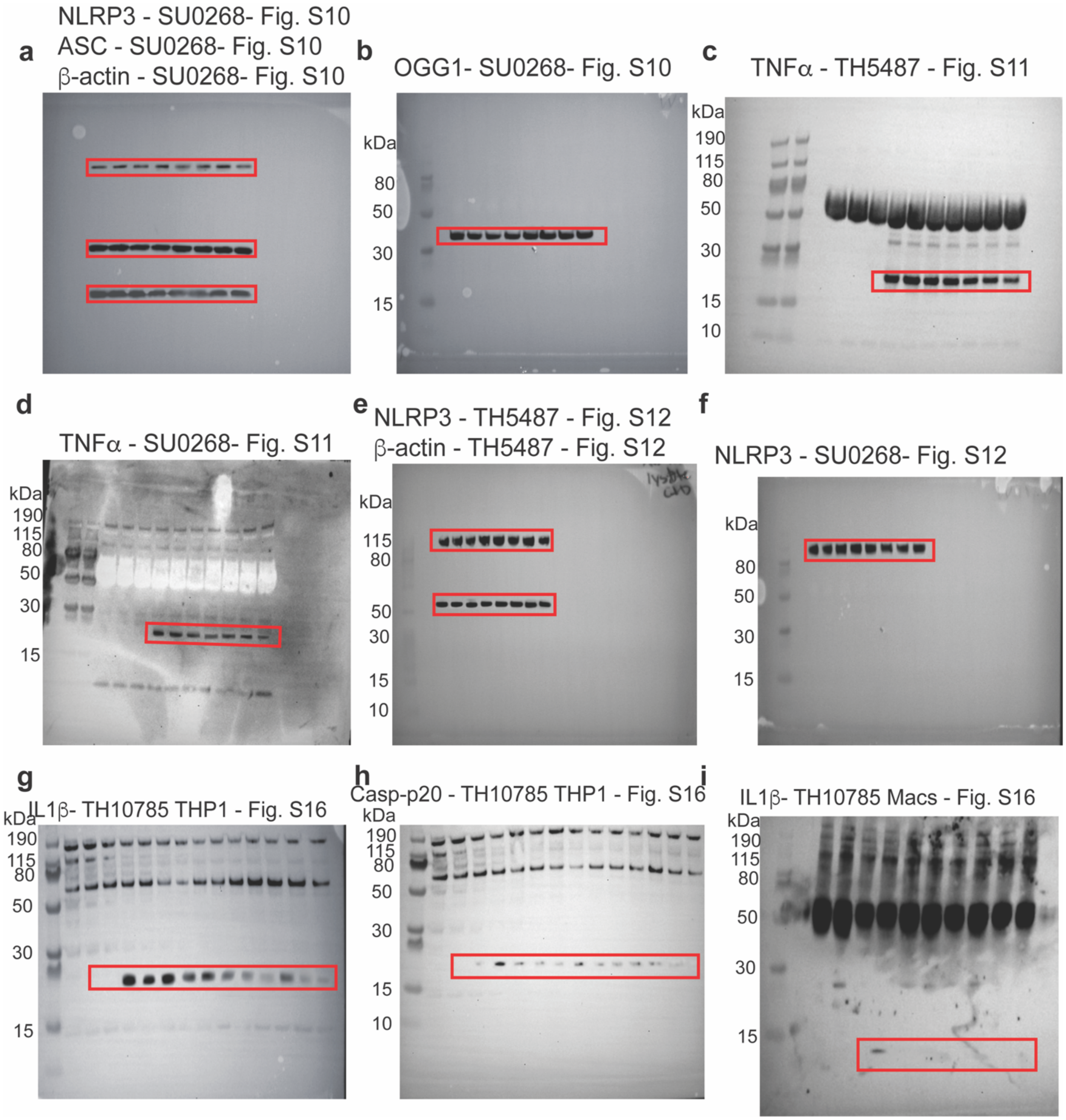

Figure S23: Uncropped blots from Figures S10, S11, S12, and S16. A-B) Figure S10 blots. C-D) Figure S11 blots. E-F) Figure S12 blots. G-I) Figure S16 blots

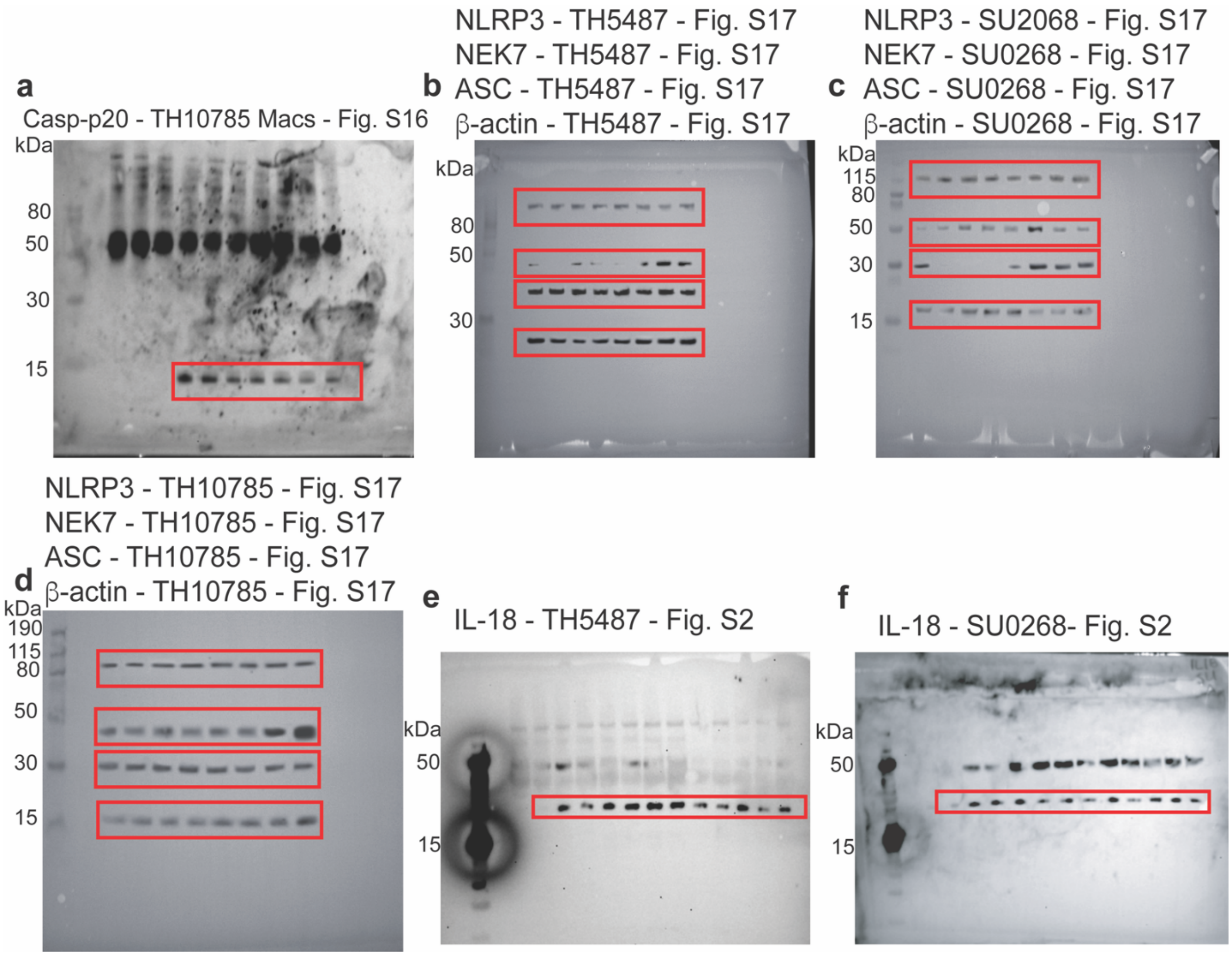

Figure S24: Uncropped blots from Figures S16, S17 and S2. A) Figure S16 blot. B-D) Figure S17 blots. E-F) Figure S2 blots.
