# Supplementary material for: TH5487 specifically targets NLRP3 in FCAS patients resistant to MCC950
Source: Commun Biol. 2026 Apr 16;9:528. doi: 10.1038/s42003-026-10008-2 (PMC13086942; doi:10.1038/s42003-026-10008-2)
Supplement: Supplementary file 2 — Reporting Summary [file 42003_2026_10008_MOESM2_ESM.pdf]

nature portfolio

Corresponding author(s): Reginald McNulty

Last updated by author(s): Feb 26, 2026

Reporting Summary

Nature Portfolio wishes to improve the reproducibility of the work that we publish. This form provides structure for consistency and transparency in reporting. For further information on Nature Portfolio policies, see our Editorial Policies and the Editorial Policy Checklist.

Statistics

For all statistical analyses, confirm that the following items are present in the figure legend, table legend, main text, or Methods section.

n/a	Confirmed	
☐	☒	The exact sample size (n) for each experimental group/condition, given as a discrete number and unit of measurement
☒	☐	A statement on whether measurements were taken from distinct samples or whether the same sample was measured repeatedly
☐	☒	The statistical test(s) used AND whether they are one- or two-sided<br>*Only common tests should be described solely by name; describe more complex techniques in the Methods section.*
☒	☐	A description of all covariates tested
☒	☐	A description of any assumptions or corrections, such as tests of normality and adjustment for multiple comparisons
☐	☒	A full description of the statistical parameters including central tendency (e.g. means) or other basic estimates (e.g. regression coefficient) AND variation (e.g. standard deviation) or associated estimates of uncertainty (e.g. confidence intervals)
☒	☐	For null hypothesis testing, the test statistic (e.g. F, t, r) with confidence intervals, effect sizes, degrees of freedom and P value noted<br>*Give P values as exact values whenever suitable.*
☒	☐	For Bayesian analysis, information on the choice of priors and Markov chain Monte Carlo settings
☒	☐	For hierarchical and complex designs, identification of the appropriate level for tests and full reporting of outcomes
☒	☐	Estimates of effect sizes (e.g. Cohen's d, Pearson's r), indicating how they were calculated

Our web collection on statistics for biologists contains articles on many of the points above.

Software and code

Policy information about availability of computer code

Data collection	NA
Data analysis	Prism, ChimeraX, ImageJ

For manuscripts utilizing custom algorithms or software that are central to the research but not yet described in published literature, software must be made available to editors and reviewers. We strongly encourage code deposition in a community repository (e.g. GitHub). See the Nature Portfolio guidelines for submitting code & software for further information.

Data

Policy information about availability of data

All manuscripts must include a data availability statement. This statement should provide the following information, where applicable:

- Accession codes, unique identifiers, or web links for publicly available datasets
- A description of any restrictions on data availability
- For clinical datasets or third party data, please ensure that the statement adheres to our policy

Requests for further information and resources should be directed to and will be fulfilled by the lead contact, Reginald McNulty, 540 Steinhaus Hall, Irvine, CA, 92697, 949-824-5541, rmcnulty@uci.edu. All unique/stable reagents generated in this study are available upon reasonable request. Data reported in this paper will be shared by the lead contact upon reasonable request. This paper does not report original code.

Research involving human participants, their data, or biological material

Policy information about studies with human participants or human data. See also policy information about sex, gender (identity/presentation), and sexual orientation and race, ethnicity and racism.

Reporting on sex and gender	Sex and/or gender were not considered in this study
Reporting on race, ethnicity, or other socially relevant groupings	NA
Population characteristics	Healthy donors and FCAS patients
Recruitment	Volunteer
Ethics oversight	IRB #180064

Note that full information on the approval of the study protocol must also be provided in the manuscript.

Field-specific reporting

Please select the one below that is the best fit for your research. If you are not sure, read the appropriate sections before making your selection.

☒ Life sciences ☐ Behavioural & social sciences ☐ Ecological, evolutionary & environmental sciences

For a reference copy of the document with all sections, see nature.com/documents/nr-reporting-summary-flat.pdf

Life sciences study design

All studies must disclose on these points even when the disclosure is negative.

Sample size	NA
Data exclusions	NA
Replication	All experiments were peformed with a minimum of three biological replicates
Randomization	NA
Blinding	NA

Reporting for specific materials, systems and methods

We require information from authors about some types of materials, experimental systems and methods used in many studies. Here, indicate whether each material, system or method listed is relevant to your study. If you are not sure if a list item applies to your research, read the appropriate section before selecting a response.

Materials & experimental systems

n/a	Involved in the study	
☐	☒	Antibodies
☐	☒	Eukaryotic cell lines
☒	☐	Palaeontology and archaeology
☐	☒	Animals and other organisms
☒	☐	Clinical data
☒	☐	Dual use research of concern
☒	☐	Plants

Methods

n/a	Involved in the study	
☒	☐	ChIP-seq
☒	☐	Flow cytometry
☒	☐	MRI-based neuroimaging

Antibodies

Antibodies used	NLRP3 Pyrin targeting (Adipogen–AG-20B-0014-C100), VDAC (Cell Signaling - #4661), b-actin (Santa Cruz - #47778), GAPDH Antibody (Santa Cruz 47724), Cleaved IL-1b (Cell Signaling #83186) Caspase-p20 (Adipogen AG-20B-0042-C100), OGG1 (Santa Cruz Biotech sc-376935), Normal Rabbit IgG (Cell Signaling #2729), ASC (Santa Cruz Biotech sc-22514-R), Pro-Caspase-1 (Cell Signaling #3866), Pro-IL-1b (Cell Signaling #12242), NEK7 (Cell Signaling #3057), P-STING (Cell Signaling #19781), IFN-b (Cell Singaling #73671), TNF-a (Cell

Signaling #3707), (Thermo Fisher), Anti-mouse IgG, HRP-linked Antibody (Cell Signaling 7076S), Anti-rabbit HPR antibody (Cell Signaling 7074S), NLRP3 polyclonal antibody (ABclonal A12694).

Validation

NLRP3 (Adipogen–AG-20B-0014-C100): Published previously in Commun Biol. PMID 37253813
VDAC (Cell Signaling - #4661): Human Reactive
β-actin (Santa Cruz - #47778): detection of β-Actin of mouse, rat, human
GAPDH Antibody (Santa Cruz 47724):GAPDH of human origin by WB, IP, IF and IHC(P)
Cleaved IL-1b (Cell Signaling #83186): Human reactive
Caspase-p20 (Adipogen AG-20B-0042-C100): Human, mouse, rat, and pig reactive by WB
OGG1 (Santa Cruz Biotech sc-376935): specific for an epitope mapping between amino acids 13-49 near the N-terminus of OGG1/2 of human origin.
Normal Rabbit IgG (Cell Signaling #2729): Endogenous Rabbbit IgG not directed against any known antigen. It was isolated from naive rabbit
ASC (Santa Cruz Biotech sc-22514-R): epitope mapping at the N-terminus of ASC of human origin
Pro-Caspase-1 (Cell Signaling #3866): Human reactive
Pro-IL-1b (Cell Signaling #12242): Human and mouse reactive
NEK7 (Cell Signaling #3057): Human Reactive
P-STING (Cell Signaling #19781): Human Reactive to residues surrounding Ser366 of human STING protein
IFN-b (Cell Singaling #73671): produced by immunizing animals with recombinant human IFN-β1 protein and recognizes endogenous levels of total IFN-β1 protein
TNF-a (Cell Signaling #3707): Human and mouse reactive, cross-reactive with monkey and pig
NLRP3 polyclonal antibody (ABclonal A12694): WB (Western Blot)Human, Mouse, Rat, Chicken, Other

Eukaryotic cell lines

Policy information about cell lines and Sex and Gender in Research

Cell line source(s): iBMDM (WT and NLRP3 KO), THP-1, Expi293 expression cells

Authentication: THP1 cell were purchased from Invivogen, Expi293 cells were purchased from Thermo Fisher and iBMDM were provided from Micheal Karin

Mycoplasma contamination: Cell lines were confirmed negative for mycoplasma

Commonly misidentified lines (See ICLAC register): NA

Animals and other research organisms

Policy information about studies involving animals; ARRIVE guidelines recommended for reporting animal research, and Sex and Gender in Research

Laboratory animals: Wild type and FCAS inducible mice

Wild animals: N/A

Reporting on sex: Sex was not considered in the study design

Field-collected samples: N/A

Ethics oversight: IRB #180064

Note that full information on the approval of the study protocol must also be provided in the manuscript.

Plants

Seed stocks: NA

Novel plant genotypes: NA

Authentication: NA
